# A bioimpedance-based monitor for real-time detection and identification of secondary brain injury

**DOI:** 10.1038/s41598-021-94600-y

**Published:** 2021-07-29

**Authors:** Alicia Everitt, Brandon Root, Daniel Calnan, Preston Manwaring, David Bauer, Ryan Halter

**Affiliations:** 1 Thayer School of Engineering, Dartmouth College, HB 8000, 14 Engineering Dr., Hanover, NH 03755 USA; 2 Neurological Surgery, Dartmouth Hitchcock Medical Center, Lebanon, NH 03766 USA; 3 Rytek Medical, Inc., 16 Cavendish Ct., Lebanon, NH 03766 USA

**Keywords:** Biotechnology, Biomedical engineering, Translational research, Techniques and instrumentation, Brain injuries

## Abstract

Secondary brain injury impacts patient prognosis and can lead to long-term morbidity and mortality in cases of trauma. Continuous monitoring of secondary injury in acute clinical settings is primarily limited to intracranial pressure (ICP); however, ICP is unable to identify essential underlying etiologies of injury needed to guide treatment (e.g. immediate surgical intervention vs medical management). Here we show that a novel intracranial bioimpedance monitor (BIM) can detect onset of secondary injury, differentiate focal (e.g. hemorrhage) from global (e.g. edema) events, identify underlying etiology and provide localization of an intracranial mass effect. We found in an in vivo porcine model that the BIM detected changes in intracranial volume down to 0.38 mL, differentiated high impedance (e.g. ischemic) from low impedance (e.g. hemorrhagic) injuries (*p* < 0.001), separated focal from global events (*p* < 0.001) and provided coarse ‘imaging’ through localization of the mass effect. This work presents for the first time the full design, development, characterization and successful implementation of an intracranial bioimpedance monitor. This BIM technology could be further translated to clinical pathologies including but not limited to traumatic brain injury, intracerebral hemorrhage, stroke, hydrocephalus and post-surgical monitoring.

## Introduction

TIME IS BRAIN when it comes to intracranial trauma^1–4^, which is both a primary cause of death and a leading cause of disability worldwide^2,5–7^. In the US and Europe alone 13 million people are living with a brain injury-related disability^8,9^, and for ischemic brain (that with limited blood supply) every *minute* of delayed treatment increases long-term disability odds^10,11^. Neurologic monitoring and timely intervention are key in managing intracranial trauma, and can be imperative to preserve healthy brain and avoid unnecessary patient morbidity or mortality.

Intracranial trauma is characterized by diffuse or focal injury and can relate to pathologies including but not limited to hematoma, traumatic brain injury (TBI), cerebral aneurysm, tumor and stroke^12–15^. Within intracranial trauma two pathophysiologic phases of injury arise: primary and secondary. Primary brain injury occurs at the initial onset of damage (e.g. cranial impact or thrombosis). However, over the following hours to days this damage can trigger a cascade of evolving secondary injuries^16–18^ which can significantly impact prognosis^6,19,20^. Among these delayed changes are cerebral edema, hematoma formation or expansion, decreased brain tissue oxygen tension and tissue ischemia^16,17,21^. Such injuries can vary in size, severity, location and presentation making monitoring (and ultimately detection) of such pathologies crucial for management of intracranial trauma patients.

Intracranial hypertension (increased pressure) is a well-established consequence of intracranial injury and leads to increases in death and poor patient prognosis^3,22,23^. Treating elevated intracranial pressure (ICP) with medical or surgical intervention mitigates the consequence of injury and improves patient outcomes; as such, monitoring ICP is the mainstay of clinical management in neurological injury^1,6,19,22,23^. However, significant limitations in ICP monitoring have motivated investigation into additional monitoring approaches to improve treatment guidance^24^.

Current bedside monitoring technology cannot distinguish between secondary injuries arising as *focal* changes in intracranial volume (ICV), such as an expanding hematoma, from those arising as *global* changes in ICV, such as diffuse cerebral edema. Further, within high-risk focal events ICP is unable to identify if the injury is *ischemic* (i.e. blocked blood flow) or *hemorrhagic* (e.g. an active bleed). Although each of these situations induce intracranial hypertension, clinical management is vastly different. Onset of secondary focal ischemia may require immediate surgical intervention^25,26^, while generalized intracranial edema may simply be treated with bedside hyperosmolar therapy (e.g. mannitol or hypertonic saline infusions). Thus, it is essential to rapidly differentiate focally- from globally-derived symptomatic intracranial hypertension^2,3,27^ and to identify the underlying etiology of acute traumatic mass lesions (e.g. ischemic or hemorrhagic) to optimally guide intervention^4,26^. The inability of bedside ICP monitoring to make this distinction in real time represents an enormous disadvantage in the clinical setting.

To help overcome these limitations, serial cranial imaging with computed tomography (CT) is frequently used to identify intracranial injury patterns and guide treatment decisions; however, acquiring repeated CTs, whether in a CT suite or with a mobile CT unit, has a number of significant drawbacks^25,28^. Maas et al. elegantly summarizes these limitations, stating that “CT can only capture momentary snapshots of the dynamically evolving process of traumatic brain injury”^16^. CTs can underestimate injury if within 3 h of onset and may lag behind actual intracerebral damage^29,30^. Suite CT scanning necessitates the patient physically leaving the intensive care unit, an act shown to exacerbate injuries^31,32^, and the time between scans (generally 6–24 h) leaves the patient vulnerable to unidentified evolving injury. Finally, serial CTs have a high cost and increase the risk of radiation-induced cancer^16,30^. With time-to-treatment linked to increased morbidity and mortality^6,33^, a continuous monitoring approach to augment and inform the use of CTs is clearly warranted.

To our knowledge, no bedside monitoring system capable of detecting, identifying and continuously tracking focal ICV changes currently exists. In this paper, we present a novel bioimpedance monitoring (BIM) system capable of measuring both impedance and ICP continuously at the bedside. Additionally, we explore the device’s ability to detect and identify onset of a secondary injury, as well as differentiate the underlying etiology (i.e. ischemic or hemorrhagic).

## Results and discussion

### Contrast mechanism

**Figure 1:**
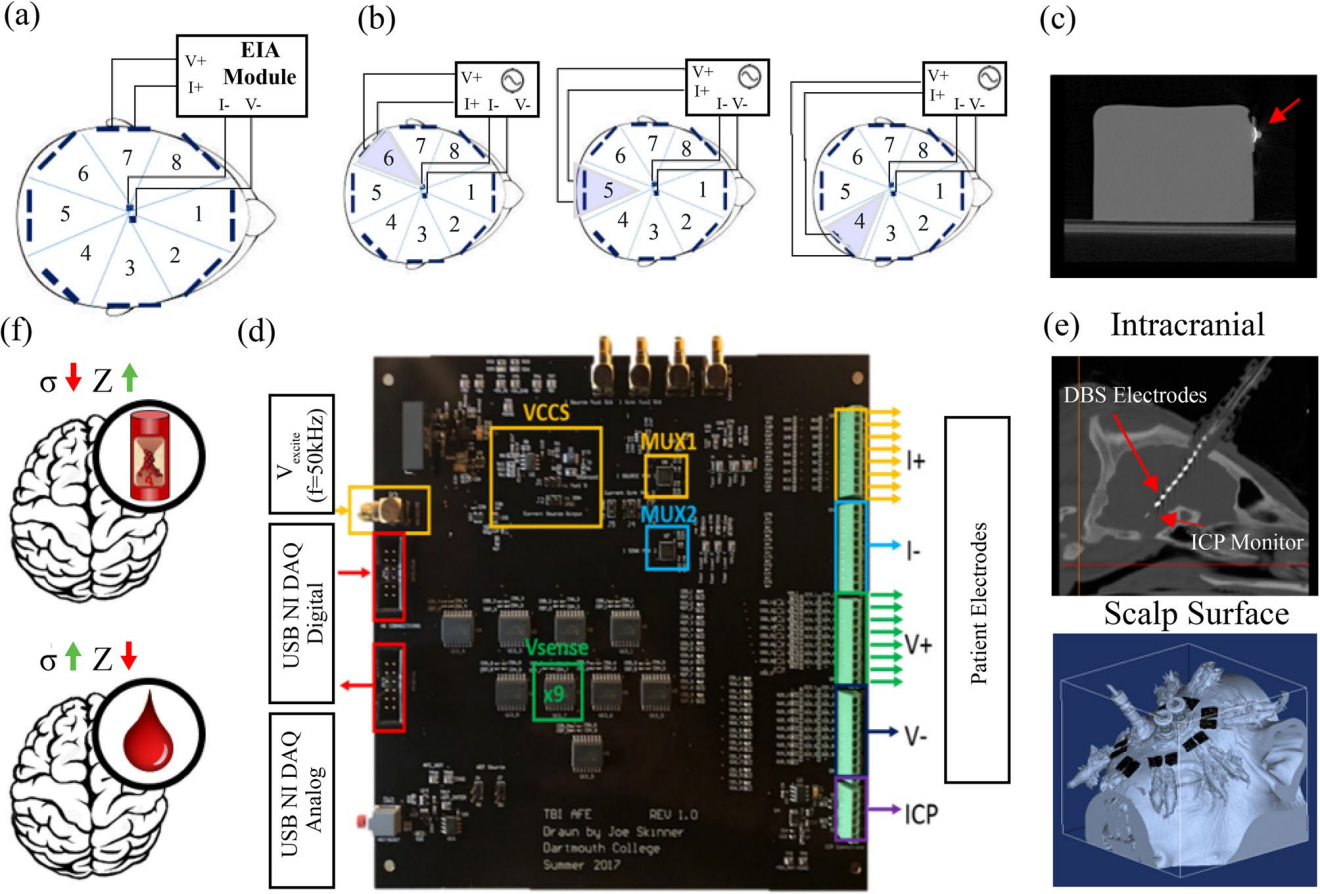
Bioimpedance monitoring (BIM) system concept, setup, testing and characterization. (**a**) Current and voltage schematic for tetrapolar (four-point) driven system. Two leads are on the scalp (I^+^ and V^+^) and two placed intracranial (I^−^ and V^−^). (**b**) Eight spatial sensitivity sectors created through rotating impedance channels and controlling the resulting electrical field. (**c**) CT scan of a gelatin phantom used to validate minimal artifact from selected biocompatible Ag/AgCl electrodes. (**d**) Custom analog front end developed for the BIM system with corresponding inputs and outputs. (**e**) Intracranial (top) and scalp surface (bottom) electrodes. The top image shows a sagittal view of the deep brain stimulation (DBS) electrode array coupled to the intracranial pressure (ICP) sensor lead in a pig. The bottom two electrodes were used for impedance sensing within the BIM system. The bottom image shows a segmentation of the surface electrodes (black) overlaid on a 3D reconstruction of a fully instrumented pig (blue). (**f**) The bioimpedance response to ischemic (top) and hemorrhagic (bottom) changes in tissue, potentially enabling differentiation between the two injury types.

Bioimpedance represents the complex electrical resistance of tissue to current flow, and is widely accepted as safe, low cost and easy to use in the clinical setting^34,35^. Impedance (Z) is comprised of resistance (R) and reactance (X) of tissue, represented by Z = R + jX. In this technology, current is injected between two electrodes (I^+^I^−^) and the induced voltage measured between two different electrodes (V^+^V^−^) (Fig. 1a). The electrical properties gauged are predominantly a function of the specific tissue types present (e.g. blood, white matter, bone, CSF) and the tissue state (e.g. normal vs. ischemic). Impedance has been validated extensively in animal models andd clinical human applications including but not limited to lung monitoring^36,37^, cancer detection^38,39^, body composition analysis and more^40,41^. In the context of intracranial monitoring, bioimpedance is sensitive to blood volume changes within the brain, as well as ischemia and stroke lesions^42–44^.

In an ischemic event cells are starved of oxygen resulting in inflammation, decrease in pH, cell swelling and rupture^45^. These responses elicit changes in the electrical properties of a particular cell, and in combination, of the tissue. Similarly, in a hemorrhagic event an active bleed leads to blood pooling or clot formation. Blood is highly conductive compared to surrounding parenchyma (~ 0.7 S/m vs ~ 0.2 S/m, respectively)^46^, altering the electrical properties of that region. When compared, an ischemic event will lower conductivity, thus increasing impedance, while a hemorrhagic event will increase conductivity and lower impedance^46,47^ (Fig. 1f).

Based on this contrast others have explored both spectroscopy and tomography of the intracranial space for diverse pathologies^43,47–49^. However, the high skull impedance acts as a significant barrier to current penetration, and has inhibited maturation of a technology utilizing the impedance contrast mechanism^50,51^. Here we build upon this foundation while exploring the novel multi-modality of impedance coupled with ICP. Through the synergistic use of both extra- *and* intra-cranial electrodes we provide a novel solution to successfully overcome the well-known limitation of current shunting. Further, by designing the BIM to integrate with standard-of-care ICP monitoring we optimally position the technology for clinical translation.

These contrasting bioimpedance responses provide a potentially specific and unique biomarker to intracranial pathology that overcomes the lack of specificity in ICP monitoring alone. We hypothesize that localized measures of bioimpedance will be sensitive to focal ICV changes, ultimately enabling differentiation of focal and global injury and lesion type identification.

### Bioimpedance monitoring: system development and characterization

System design requirements needed for secondary brain injury monitoring are outlined in Supplementary Table 1. The primary design criteria included 80 dB SNR, 99% accuracy, 100 kHz bandwidth, and multi-channel data acquisition. Further, the BIM system was designed to be compatible with CT for validation purposes.

Our BIM uses a tetrapolar (four-point) measurement configuration to provide broadband impedance acquisition and maximize sensitivity. Two drive electrodes inject current (I^+^ and I^−^) via a Howland-based voltage-controlled current source^52^ and two pick-up electrodes measure the induced potential (V^+^ and V^−^), as shown in Fig. 1a. Multiplexing electrode configurations facilitate multi-channel monitoring (Fig. 1d) such that the central intracranial electrodes (I^−^ and V^−^) remain fixed while the source electrode pair (I^+^ and V^+^) rotates about the scalp. This enables localized sensitivity in the form of eight impedance sectors (Fig. 1b).

Biocompatible materials were selected for all device components which come into contact with the animal. Eight circumferential pairs of Ag/AgCl tab surface electrodes serve to source current and measure voltage (I^+^ and V^+^); these electrodes were chosen to minimize CT artifact (Fig. 1c). Additional imaging validation of selected materials is provided in Supplementary Figure 1. Clinically approved multi-channel deep brain stimulation (DBS) leads are used as intracranial electrodes to sink current and voltage (I^−^ and V^−^) (Fig. 1e). Directly coupling our internal electrodes to a standard ICP sensor enables this hybrid-component to be introduced through the same procedure currently employed to manage patients with severe intracranial trauma (i.e. ICP catheter introduction through a skull-placed burr hole and cranial bolt). Clinical feedback regarding this design has been highly positive with no concerns for practical implementation.

A custom analog front end (AFE) provides multiplexing between channels (Fig. 1d) and successfully captures broadband (10 Hz–100 kHz) impedances with sufficient accuracy (99.7%), precision (SNR = 84.28 dB), and temporal stability (< 0.03% CV) for an intracranial monitoring application (Supplementary Figure 2).

### Model selection

When considering secondary brain injury, a volume controlled focal increase and decrease in impedance was needed to (1) validate the BIM’s detection of contrasting impedance mechanisms, (2) quantify volume sensitivity to focal changes, (3) differentiate focal from global-derived ICP changes and (4) explore the BIM’s coarse ‘imaging’ capabilities through localization of a mass effect. Intracerebral hemorrhage is well established as a high-risk focal trauma and is typically modeled through intraparenchymal injection of low impedance autologous blood^53–56^. Additionally, Fogarty catheter balloons are a well-established method for inducing controlled intracranial hypertension in an animal model^57–59^. Specifically, they represent a localized increase in volume and impedance as they are gradually inflated, serving to model a high-impedance intracranial mass effect^60^. Both injury mechanisms (balloon inflation and autologous blood injection) used for the in vivo swine model produce focal volume change with subsequent elevation in ICP; however, the changing impedance characteristics differ (Fig. 1f). For global injury an injection of mannitol and the capture of brain death represented significant ICP change without a focal ICV change. These induced injuries enabled controlled validation of the novel BIM technology on key secondary pathologies.

### Surgical system and integration

All animals (n = 9) were instrumented with an ICP monitor (Mikro-Tip Catheter Pressure Transducer, Millar, TX), 1.2 mL intracranial catheter for mass effect and hematoma (Fogarty Thru-lumen Embolectomy Catheter, Edwards, CA), 150 µL intracranial catheter for compliance (Fogarty Arterial Embolectomy Catheter, Edwards, CA), monitoring system for vitals (MP150, Biopac Systems Inc., CA), and the novel BIM. Frameless stereotactic guidance with the Stealth AxiEM system (Medtronic Navigation, Louisville, CO) allowed for targeted placement of each intracranial implant. All instrumentation was arranged in the bore of a CT scanner to enable continuous CT acquisition and provide imaging confirmation of focal ICV changes.

**Figure 2:**
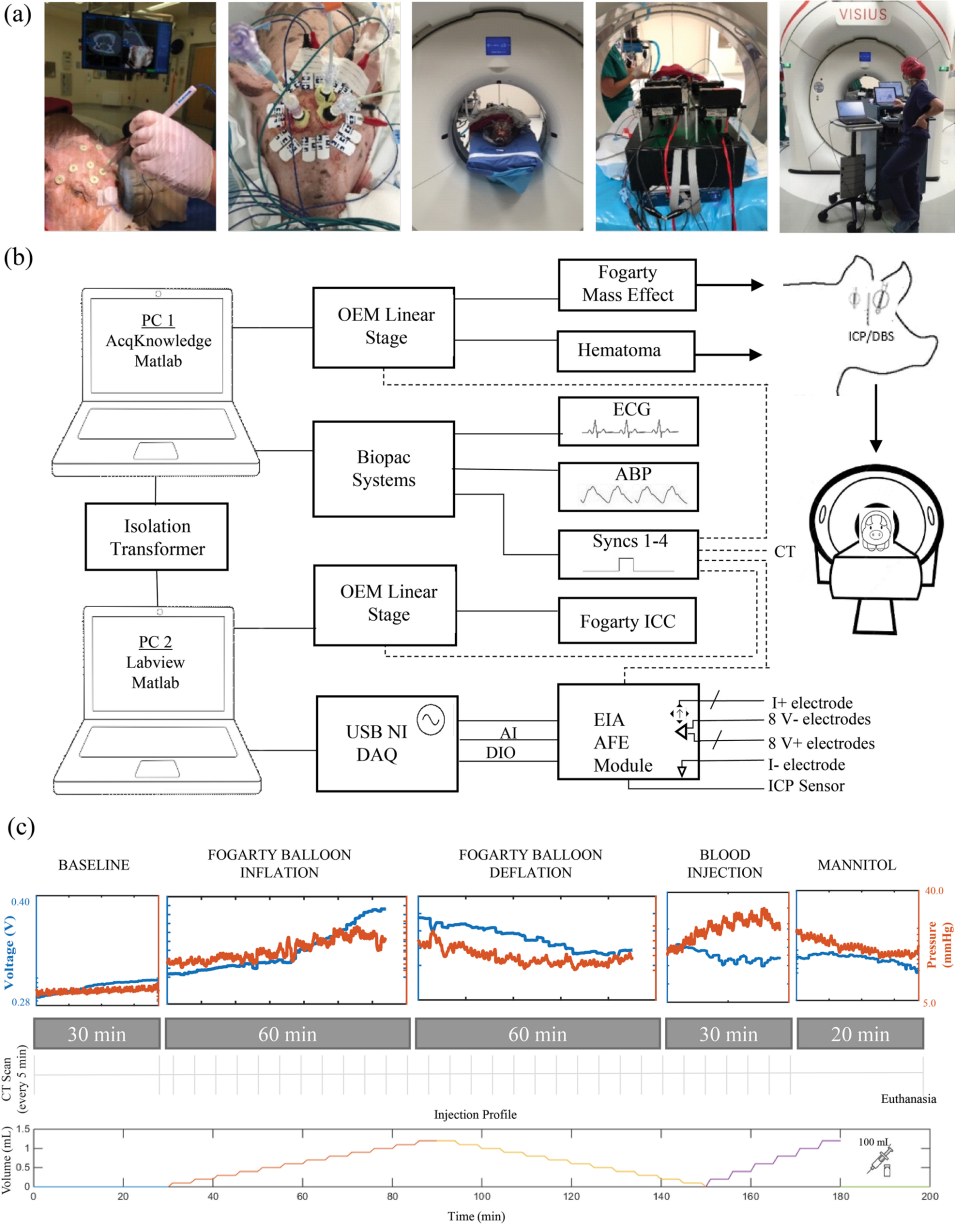
Surgical implementation and system diagram. (**a**) From left to right: AxiEM navigation and fiducial registration being used to enable precise neurosurgical instrumentation. Top-view of an instrumented pig displaying the scalp surface electrodes, the cranial bolts, the electrode leads, and the three Touhy–Borst adapters securing the two Fogarty catheters and the DBS/ICP lead. The pig successfully setup within the bore of a CT scanner. The instrumentation located at the head of the pig including two syringe pumps, the AFE and the data acquisition unit (DAQ). Lastly, the full system including pig, electrodes, control computers and all supporting hardware in the operating room. (**b**) Block diagram showing the relationship between all devices in a full test setup, including the electrical impedance acquisition (EIA) system. (**c**) Protocol used for testing of BIM system with each phase broken down including the two injury types, timings and volumes. Orange and blue traces are ICP and Z, respectively, from a consistent channel across Pig 1. Volume changes can be seen clearly as discrete steps in the ICP curves.

Each system was synchronized using the Biopac digital I/O. The CT ‘acquire’ button was instrumented with a custom 3D printed momentary-switch used to drive a digital line to the Biopac System (Supplementary Figure 3). Computer-actuated syringe pumps provided precise volume control for each intracranial catheter. Surgical implementation of the system is shown in Fig. 2a, and a block diagram of the full data acquisition configuration is detailed in Fig. 2b.

**Figure 3:**
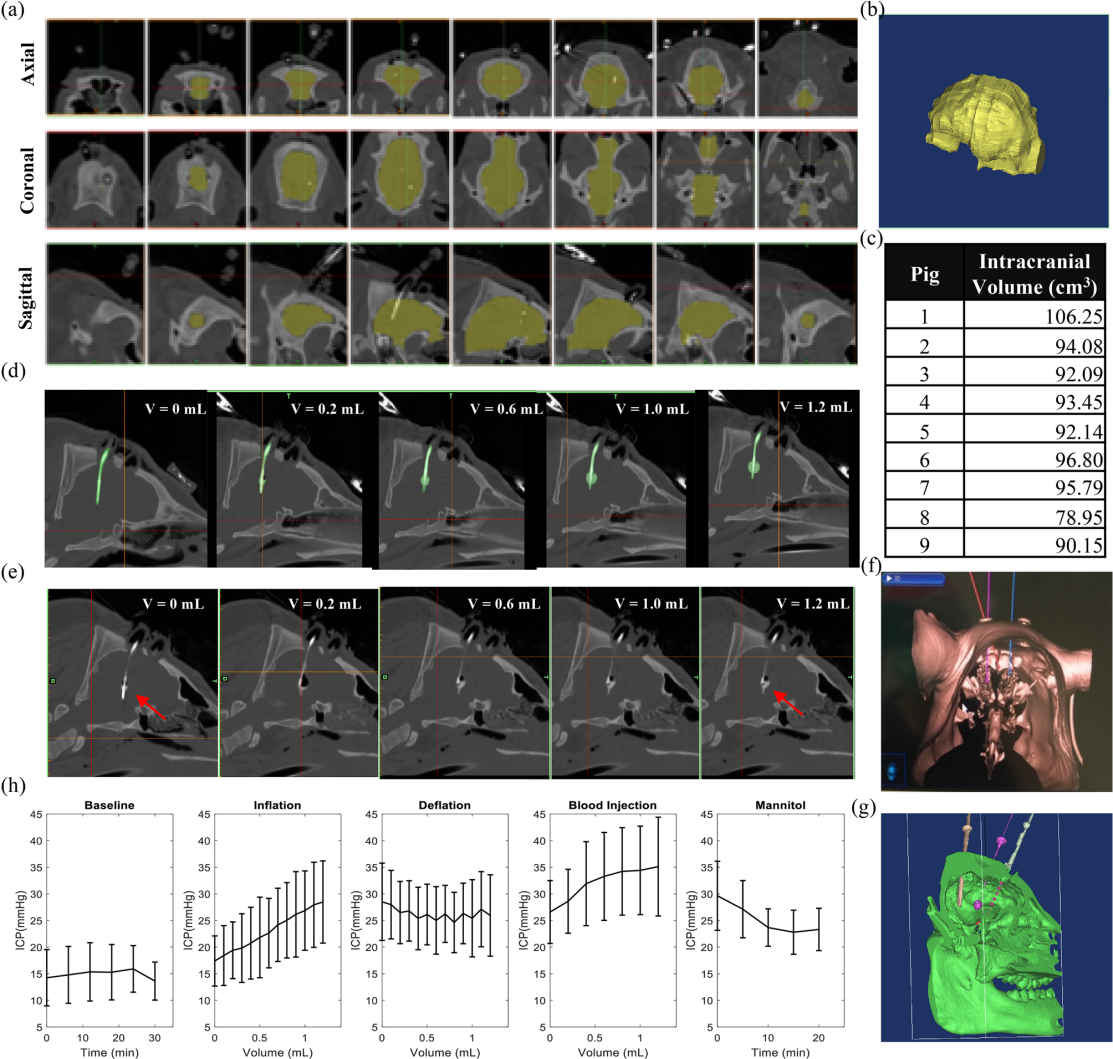
Computed tomography imaging integration and precise capture of evolving intracranial injury. (**a**) Axial, sagittal and coronal views of cross-sectional masks of the brain created in Mimics. Images spatially advance left to right. (**b**) 3D segmentation of the same brain generated from interpolation between masks. (**c**) Segmented intracranial brain volumes for all pigs. (**d**) oronal view of inflating Fogarty balloon from 0 to 1.2 mL volume. (**e**) Coronal view of injected autologous blood from 0 to 1.2 mL volume. **(f)** Stealth navigation surgical plan used for precise placement of the three intracranial instruments, enabled through registration with pre-operative images. (**g**) 3D reconstruction of post-op intracranial space with three catheters in place and segmented for localization. (**h**) ICP response summaries across pigs (n = 9) for each protocol phase. Error bars represent  ± one standard deviation. Note the ICP increases as volume increases for balloon inflation and blood injection.

After instrumentation the pig rested at baseline for 30 min. Balloon inflation, balloon deflation, autologous blood injection, treatment with mannitol and euthanasia all followed baseline, respectively (Fig. 3a). CTs were acquired every 5 min and blood gasses collected at the beginning and end of each event (Fig. 2c). The system successfully recorded ICP and Z. Traces of raw data collected during each event are displayed in Fig. 2c. ICP and Z both increased for balloon inflation, decreased for balloon deflation, and had varied responses for blood injection—which are analyzed in more detail in ‘Detecting secondary brain injury’ section. Blood gasses were stable through anesthesia and ventilation (Supplementary Figure 4), and no animals suffered any significant respiratory or metabolic distress that would have induced confounding effects on brain or body physiology, such as hypoxia (Supplementary Table 2).

**Figure 4:**
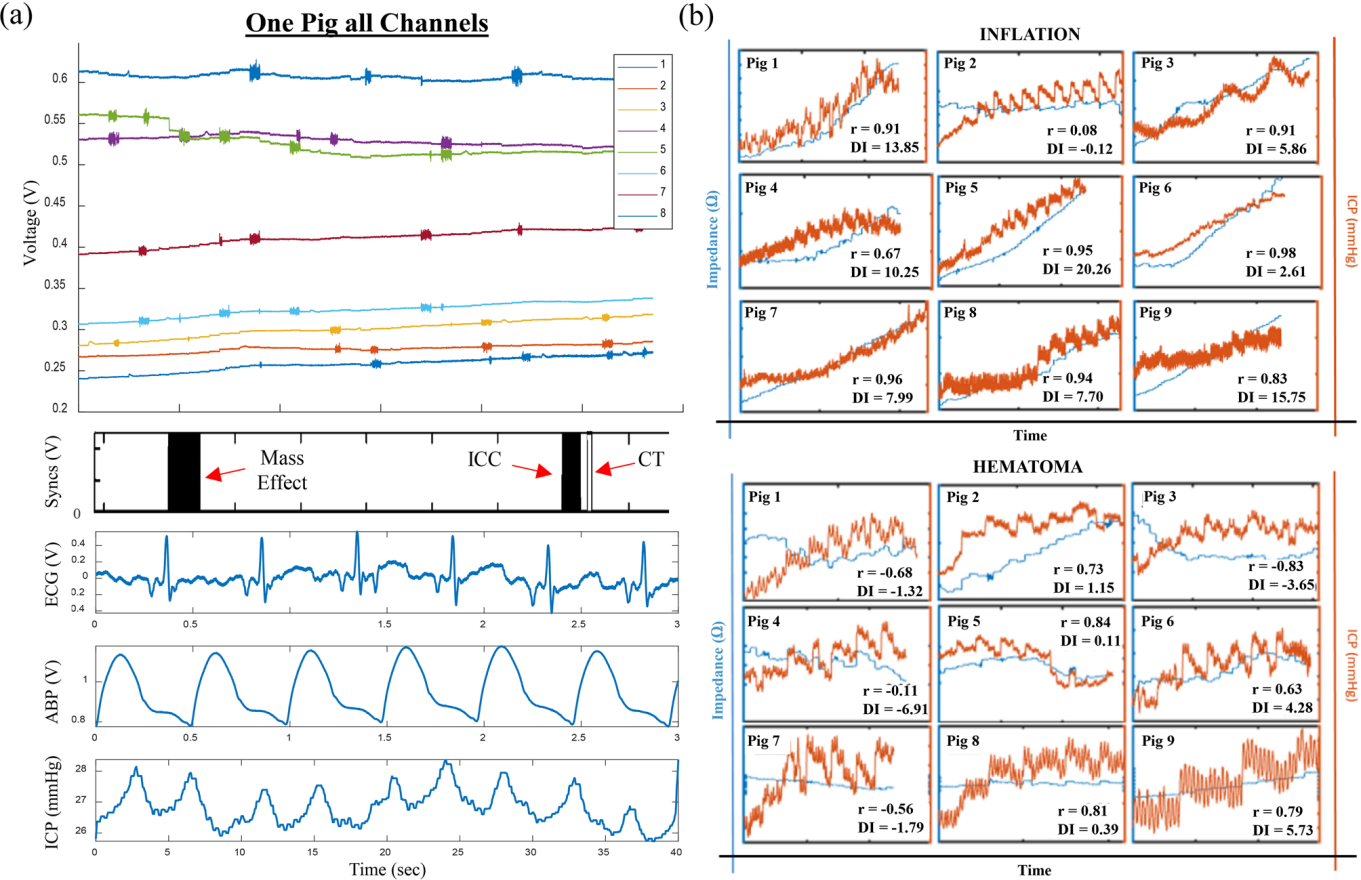
Initial differences in impedance behavior between inflation and hematoma. (**a**) All collected traces from one pig: eight impedance channels and all Biopac physiologic signals. Sync Traces show linear stage activation for each Mass Effect injection, spaced 5 min apart. Each ICC check occurs 2 min following Mass Effect. CT acquisition followed ICC, with precise timing of the manual acquisition captured through a custom sync button. All Biopac signals are shown in seconds. High noise sections on impedance channels are associated with data recorded during active acquisition of a CT and these artifacts are removed prior to data analysis, as described in Supplementary Method 1. (**b**) ICP (orange) and impedance (blue) for all animals and both injuries across a single electrode. Pearson’s correlation coefficients and DI represented for each animal for the presented trace. Impedance y-axes are normalized within each animal between injury types.

### Intracranial imaging and model response

Serial CT scans successfully captured the evolving state of the intracranial space and enabled ancillary functions (e.g. surgical navigation and post-operative segmentation) needed to validate detection of secondary brain injury. One potential limitation to a volume-controlled study is that the same ICV change may represent varied ‘trauma’ to each subject (i.e. %ICV change is dependent on baseline ICV). Therefore, the baseline brain volume was extracted through use of Mimics imaging segmentation software (Materialise, Leuven Belgium). Figure 3a shows cross-sectional slices in sagittal, axial and coronal views of an example intracranial image stack. Such slices are reconstructed to define the 3D volume (Fig. 3b), and brain volumes are computed for each pig (mean volume: 93.3 ± 7.12 cm^3^) (Fig. 3c). Further, evolution of injury was tracked in image space for both mass effect (Fig. 3d) and intracerebral hemorrhage (Fig. 3e). Autologous blood was mixed 5:1 with contrast (Visipaque) to enable visualization of the hemorrhagic injury—an essential step as unanticipated variability in the hemorrhage model was illuminated through CT assessment (Fig. 6b). Lastly, surgical guidance enabled tailored placement of intracranial instrumentation to each pig through initial scanning, registration and pre-operative surgical planning. Figure 3f,g show this surgical plan and the comparable post-operative reconstruction of the inclusions, respectively.

Fogarty balloon inflation monotonically increased ICP using controlled volume increments, matching previous findings of balloon-based ICP control^57^. Further, both injury types, balloon and hematoma, increased ICP—demonstrating that ICP elevation is independent of the underlying mechanism of injury (Supplementary Figure 8). Administering mannitol reliably decreased ICP, also matching expectations (Fig. 3h). As Fig. 3 shows, there was variability amongst pigs, however all followed expected trends.

### Detecting secondary brain injury

#### Identifying and differentiating intracerebral hemorrhage and mass effect

Bioimpedance data was recorded from all eight channels for each pig (Fig. 4a). Figure 4b shows filtered bioimpedance and ICP traces for all pigs and both injury types. Raw impedance channels were pre-filtered to ensure accurate assessment of impedance changes by separating injury response from physiological (e.g. blood compromised electrode) and electrical (e.g. CT-induced DC shift) artifacts (see Supplementary Method 1). During blood injection Pig 5 suffered intracerebral herniation due to the elevated ICP (note sudden sharp ICP decline in Fig. 4b), and thus is excluded from the following hematoma analysis.

As expected, Z monotonically increased with volume (and ICP) during balloon inflation (mean Pearson’s correlation of *r* = 0.803). For hematoma, Z was much more variable as the higher conductivity (lower Z) focal event contrasted the rising ICP (mean Pearson’s correlation of *r* = 0.098). The difference between coefficients supports the hypothesis that the BIM is sensitive to underlying focal injury etiology.

One would have expected the highly conductive blood (~ 0.7 S/m^46^), as compared to the surrounding parenchyma (~ 0.2 S/m), to drive the impedance down as volume increases, such as occurred with conductive inclusions in phantoms (Supplementary Figure 5). However, the large number of cases (5 of 9) with positive correlation suggest that, once in the brain, the interplay between impedance, volume and pressure is much more complex. As volume increases so does pressure, increasing density of the surrounding parenchyma, suggesting that impedance behaves as a function of both pressure and volume, or $Z\to f(P,V)$. We define a discriminatory index (DI) as the ratio of impedance change to pressure change, in an attempt to quantify this relationship:

$$DI=\frac{\Delta Z}{\Delta ICP}$$

**Figure 5:**
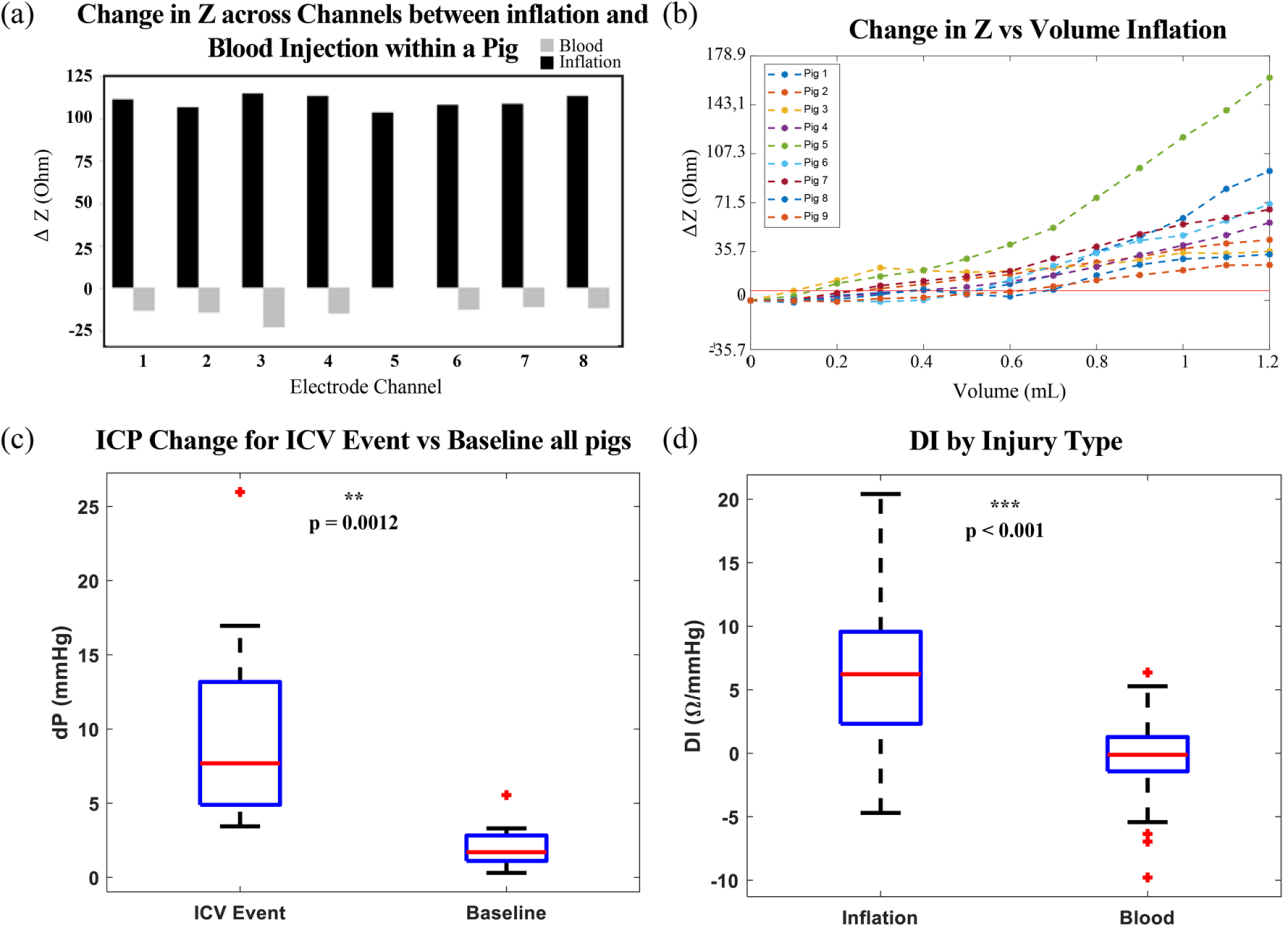
Identification and differentiation of secondary brain injury. (**a**) ΔZ compared between injury types across all channels within a single pig. Missing channel (5, blood) is a filtered trace. (**b**) Change in impedance from baseline of de-trended volume balloon inflation. Threshold for detection determined from SNR with a 10 × safety factor (7.1 Ω) and represented by the horizontal red line. Impedance successfully detected ICV change in all nine pigs. (**c**) Change in ICP successfully differentiates a volumetric event from baseline, however cannot discriminate between injury type (Supplementary Figure 8). (**d**) Discriminatory Index significantly differentiates the type of event (injury) occurring.

The DI was computed from filtered data for the two simulated injury types in Fig. 4b. Figure 5a shows the ΔZ for each channel during inflation within a single pig. The expected channel-to-channel variability presented here arises from the unique anatomical location of each channel. In addition, the location of each channel in relation to the injury varies—a dependence that we capitalize on for localization in “Focal and global differentiation” section .

In 9/9 pigs, mean impedance change from a de-trended baseline successfully detected ICV change (0.38 mL ± 0.19 mL) (Fig. 5b). In a clinical setting should the lesion location be known (i.e. based on initial CT scans), the closest channel alone also successfully detected the volume change in 9/9 pigs (0.39 mL ± 0.24 mL), see Supplementary Figure 6. Lastly, should a baseline collection not be possible, ΔZ without de-trending also yielded volume detection in 8/9 pigs (0.29 mL ± 0.15 mL), see Supplementary Figure 7. However, in the clinical application of secondary injury detection, a continuously updating global trend should always be possible. While detection occurred at a smaller volume without de-trending, one must be cautious in interpreting this observation, as global rises in impedance associated with electrode settling may falsely yield a ΔZ independent of volume change if within the first hour.

ICP alone was unable to differentiate between a hemorrhagic or ischemic event (Supplementary Figure 8), however could identify that an event was occurring when compared to the baseline collection (Fig. 5c). With the addition of the BIM, DI was successfully able to differentiate between inflation and hematoma across all pigs by a within-subjects mixed model (*p* < 0.001) (Fig. 5d). Should all elements within each pig be averaged and then compared between injury type, DI also significantly differentiates between the injuries by a paired student’s t-test (*p* = 0.0013) (Supplementary Figure 9). These results validate the original hypothesis, and further substantiate the potential of the BIM to both detect onset of secondary injury and identify injury type.

#### Focal and global differentiation

**Figure 6:**
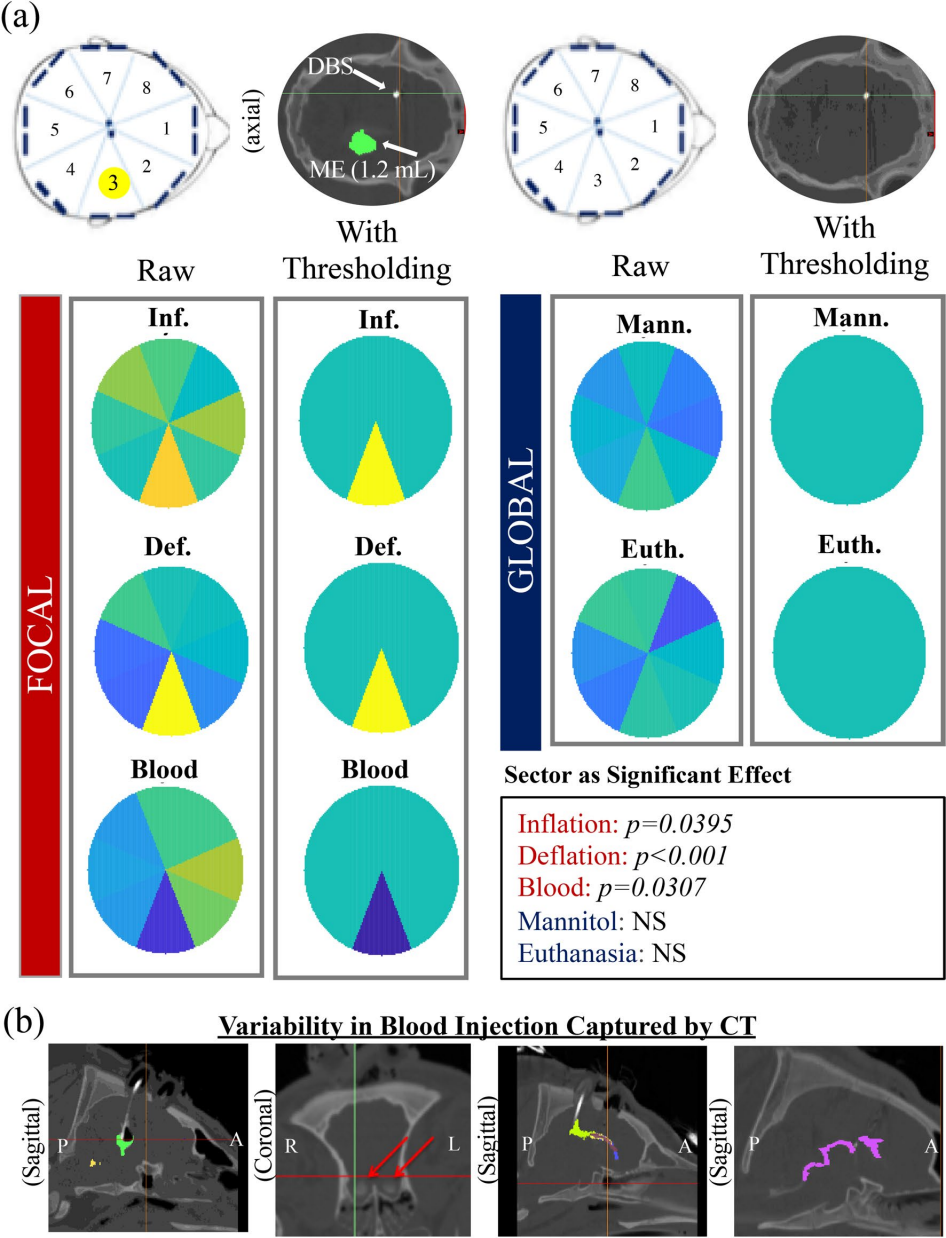
Localization between focal and global events. (**a**) Colormaps of normalized change in impedance (nΔZ) for each event across all pigs (caxis: 0–1). Raw maps show the mean nΔZ for all elements, while threshold maps display a discrete differentiation between both focal and global events and between ischemic and hemorrhagic injuries. A threshold approach was heuristically determined with thresholds of nΔZ < $\frac{1}{3}$ and nΔZ > $\frac{2}{3}$. Should any element satisfy one of these thresholds, the binary map identifies the minimum or maximum element as the discrete focal injury element for hemorrhage or model ischemia, respectively. Any event with all nΔZ values between $\frac{1}{3}<{{\rm n}}\Delta {{\rm Z}}<\frac{2}{3}$ were categorized as global. Inclusion was located in Element 3. Only focal events presented an element’s ‘sector’ as a significant effect in a mixed model. (**b**) Display of some of the variabilities present in the hematoma model captured by the CT scan, competing with focal localization ability. From left to right: induced air during blood injection, pooling of blood in the olfactory ventricles (unique to pigs), anterior blood tracking, posterior ventricular tracking down to the spinal cord (most common).

In addition to identifying focal injury etiology, the ability to differentiate elevated ICP as derived from a focal or global event could have transformative patient and clinical intervention implications. Phantom experiments validated the system’s ability to detect a volume change of less than 5 mL, correctly localize inclusions to a specific sector, and to differentiate conductive and insulative inclusions (Supplementary Figures 5 and 10). Once in vivo, impedance channels in each pig were normalized by the highest channel (scale of 0–1). This served to elucidate heterogeneity of the intracranial space within each pig. For normalized ΔZ (nΔZ) all focal events (inflation, deflation and hematoma) showed channel as a significant fixed effect of a mixed model across all pigs (*p* = 0.0141), while all global events (mannitol and euthanasia) had no significance (Fig. 6a). Threshold color maps for nΔZ show Element 3, where the inclusion is located, to be most prominent in response to the volume change, matching phantom results. These results support both detection and potential localization of focal injury, and were further explored via quantitative thresholds.

A powerful attribute of the study was precisely imaging intracranial behavior at each injury through acquisition of serial CT scans. These images captured unanticipated limitations of the model and helped in interpreting the recorded impedance signatures. For instance, while blood injection is a well-established model for hemorrhage^53,54^, the CTs surprisingly showed that not all of the injected blood stayed local, but instead tracked away posteriorly (Fig. 6b). While unexpected, a localized pool at the injection site was still maintained (Supplementary Figure 11), supporting the model’s use in representing an injury; further, the hybrid blood behavior demonstrates the robustness of the BIM’s ability to differentiate etiology in a less controlled clinical presentation of injury. However, for the analysis as a true focal event it is confounded by simultaneous blood diffusion. Similarly, while mannitol acts globally it has been shown to preferentially diffuse water from areas of higher injury (e.g. focal area around mass effect), also identifying it as a hybrid event^61^. Fortunately, other aspects of the protocol (i.e. deflation) represent focal change, while euthanasia captures the commonly studied event of brain death^62^ (global change). ICP identified euthanasia and deflation as intracranial events (*p* < 0.001), supporting their use for analyzing the BIM’s ability to differentiate focal from global etiology (Supplementary Figure 12).

**Figure 7:**
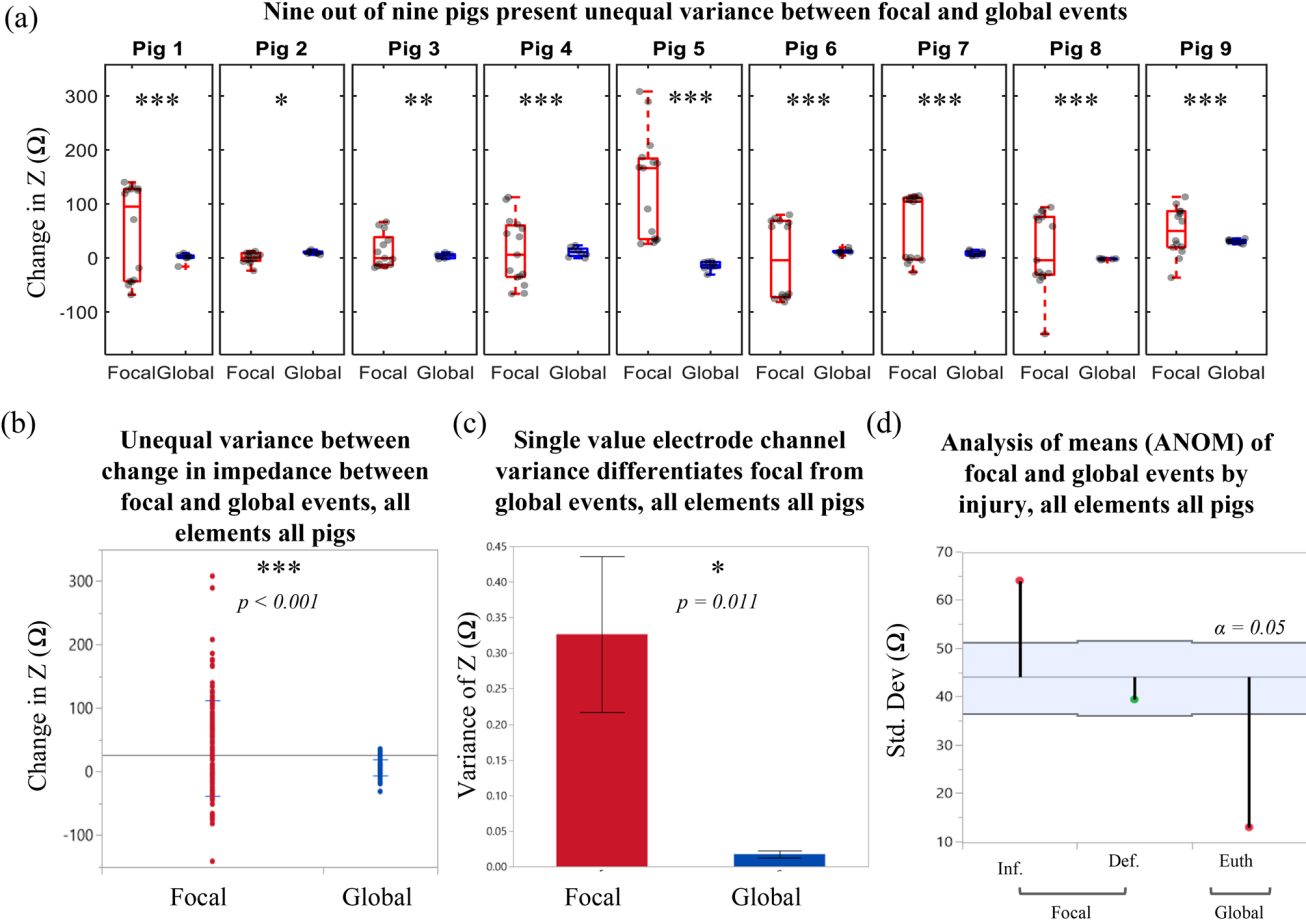
Focal versus global differentiation within and across pigs. (**a**) Change in impedance between focal (balloon volume change) and global (euthanasia induced brain death) intracranial events within each pig. Nine out of nine pigs showed significant difference in variance (Levene’s) between focal and global events (*p < 0.05; **p < 0.01; ***p < 0.001). (**b**) Comparison of variance between change in Z for all elements within all pigs between focal and global events shows significant difference. (**c**) If all elements are averaged into a single-value variance within each pig for each event, the single value variance significantly differentiates focal from global injury (Welch’s t-test to account for unequal variance). (**d**) Analysis of means for the standard deviation of impedance change for each event of the protocol shows differentiating trends and significant differences from the mean.

Significant differences in sector variance were observed between focal and global events, with focal defined as balloon volume changes and global as euthanasia induced brain death (Levene’s *p* < 0.001, Fig. 7b). That is, across all pigs for the eight electrode pairs, when a focal event occurred the variance across channel measurements was significantly higher than when global. Further, *within* each pig there was significant difference in variance in 9/9 pigs between focal and global events (Fig. 7a). Injury type (focal vs global) was found to be a significant effect (mixed model, *p* < 0.001) across all pigs and impedance changes. A single value variance calculated for each pig from each 8-electrode channel set significantly differentiated between focal and global events (Welch’s t-test to account for unequal variance, *p* = 0.011) (Fig. 7c).

An analysis of means (ANOM) of the standard deviation of the impedance changes represents an additional quantitative metric to augment nΔZ (Fig. 7d). The ANOM shows significant difference from the mean for both inflation and euthanasia based on a 44.1 Ohm threshold. Deflation was not significantly different from the group mean; however, this can be explained by the pressure response for deflation (Fig. 3h). If we consider inflation vs euthanasia, our most controlled events, we retain significant difference in variance across all pigs (*p* < 0.001, Supplementary Figure 13), *within* pig statistically significant variation of 5/9 pigs (Supplemental Figure 14), and higher variance for inflation than euthanasia in 9/9 pigs. The reduction of in-pig statistically significant detection (our least powered scenario) is an important recognition of our limited number of samples (n = 8 channels). In future work additional electrode channels will help to increase the samples and spatial resolution across the brain.

Currently there is no way to identify the onset of a secondary brain injury outside of the limited ICP metric. The novel BIM presented here exhibits capabilities to detect onset, differentiate injury type and distinguish focal from global events. While limitations were present in the minipig model, including the wide range of baseline ICPs, non-isolated conductivity injury model (e.g. air injection and blood diffusion), complex physiologic responses (e.g. comorbidity trauma response observed from instrumentation alone), and lack of statistical randomization (discussed further below in methods), each of these complexities robustly captures the intricacies of human monitoring—and ultimately are a strength of the in vivo model, further highlighting the BIM’s capabilities beyond an in vitro setting. Now it can be conceptualized that in the clinic the BIM would be placed around the head and used to continuously monitor each element. Changes to volume of initial trauma, evolving secondary injury, intracranial heterogeneity and global trends could all be reported as essential metrics for patient management. Further, upon BIM-based detection of an event, differentiation of type and identification as focal vs. global, an alert will be triggered and the patient immediately taken for an investigatory or pre-operative CT scan, something not possible with current ICP-based monitoring strategies alone.

## Conclusion

This paper presents a novel monitoring system for detecting secondary brain injury. The system was successfully designed, developed, characterized, and evaluated in an animal model. The system proved capable of detecting an intracranial volume change, differentiating that injury as high impedance (e.g. ischemic) or low impedance (e.g. hemorrhagic) and separating global from focal intracranial changes. These results demonstrate for the first-time the potential to detect the onset of secondary brain injury and differentiate the etiology in real-time at the bedside.

## Outlook

While outside the scope of this paper, it is important to emphasize the broader potential impacts of this technology. Monitoring and detecting intracranial volume changes is not isolated to severe brain trauma; other conditions, including TBI, stroke, hydrocephalus, epilepsy and extra-axial hematomas also experience localized volume change events (e.g. ischemic and hemorrhagic stroke), suggesting that a variant of this BIM system might be extended to a broad range of clinically relevant pathologies.

## Methods

### Study design

Our study was designed to test the hypothesis that a bioimpedance-based monitor could detect focal intracranial volume changes of a significant clinical threshold, differentiate high impedance from low impedance injury, and localize that injury in the cranium. Based on clinical consultation an original objective was to measure a minimum intracranial volume change of 500 μL. Initial imaging analysis and phantom data suggested a volume change of 500 μL to be associated with a respective voltage change (linear relation to impedance) of ~ 0.003 V and a standard deviation ~ 0.002 V. Assuming a matched pairs difference of means analysis, we required a minimum sample size of 8 animals to demonstrate significant differences with a power of 0.95 (assuming alpha = 0.05 and no-injury results in mean difference = 0 V). No data were excluded prior to pre-processing and CT review. During pre-processing, data was filtered in accordance to Supplementary Method 1 and “Data exclusions and filtering” section below. All replicate experiments were successfully reproduced across all pigs. Repeatability was controlled by the same neurosurgeon performing the procedure on each animal. Additionally, surgical guidance (AxiEM Stealth) permitted precise navigation and placement of catheters during animal instrumentation. Continuous CT imaging permitted real-time validation of the intracranial state and induced injury. Lastly, three preliminary pigs (not discussed) were used prior to initiating data collection to optimize surgical approach and ensure a repeatable, controlled model. Randomization was not relevant to this study as all animals underwent the same procedure. As all animals were of the same cohort no blinding to cohort was required. Within each localization analysis the color maps were blind to the location of the inclusion, and later validated against CT scans. Similarly, during system characterization the phantom experiments which (1) tracked a moving inclusion and (2) differentiated high Z from low Z focal changes were blind to the location and conductivity of the inclusion until validation of the results. Other statistics (e.g. descriptive statistics and difference of means) required knowledge of which event was occurring and were not relevant to blinding.

### Experimental animal selection and care

Nine Yucatan miniature swine were used for the protocol. Swine were considered the optimal animal for this study given their large size and comparable anatomy to humans, specifically, gyrencephlic brains and similar white to grey matter ratio (Supplementary Note 1). All animals were female (n = 6) or castrated male (n = 3), 25–30 kg and 0.4 years of age. Swine were bred in a research specific facility, tested pathogen free and approved for use in the Center for Surgical Innovation (Dartmouth Hitchcock Medical Center and Dartmouth College)—a specialized operating suite with intraoperative CT scanning capabilities and access to full human surgical amenities including image-based surgical navigation.

All animal procedures were conducted according to the NIH Guide for the Care and Use of Laboratory Animals. The protocol was approved by Dartmouth’s Institutional Animal Care and Use Committee, and adhered with ARRIVE guidelines.

### Bioimpedance monitoring system

The bioimpedance monitoring (BIM) system consists of a National Instruments (Austin, TX) multi-channel data acquisition module (USB-6363) controlled by LabVIEW. A custom analog front end (AFE) printed circuit board was designed to interface to the USB-6363. The AFE utilizes 16 surface electrode channels. Ag/AgCl scalp electrodes (Kendall Care, EK310 Electrodes, NY) were selected to minimize any CT artifact. Two most distal contacts on a deep brain stimulator (Medtronic, DBS Lead 3387, MN) were used as our intracranial electrodes. The pair of intracranial electrodes on the DBS device are adjacently coupled to an intracranial pressure sensor (Fig. 1e). A Howland voltage controlled current source (VCCS) provided precision current control over the range of 0.5–5.0 mA. For this application, the VCCS was programmed to inject a 2.3 mA_pp_ sinusoidal current at 50 kHz. Current and voltage were sampled from each channel at 50 kHz and the amplitudes extracted using a matched filter^63^. Impedances were computed as the ratio of voltage to current at a rate of 50 Hz with averaging of 1000 samples per data point. Each BIM channel records impedance for ten seconds before being switched to the next channel.

The system was characterized through a series of discrete and in vitro tests. For SNR evaluation, four discrete resistance loads (10 Ω–2 kΩ) were each measured continuously for a half hour and the SNR was computed from the standard deviations and mean of each data set (SNR = 20log_10_(mean/std)). For accuracy characterization, a linear calibration curve was fit across five resistor measurements (10 Ω–2 kΩ) and two independent resistor values within range but not of the discrete values used for calibration were predicted via the BIM and compared to their benchtop measured values. Frequency bandwidth was confirmed through assessment of voltage drop as a function of load and frequency, and temporal stability was defined as the coefficient of variation (CV) over a ~ 3 min time window.

### Anesthesia, analgesia, and monitoring

The pigs were premedicated with midazolam 0.5 mg/kg im and ketamine 22 mg/kg im. The pig was endotracheally intubated and general anesthesia maintained with 1–3% inhaled isoflurane and Fentanyl 20 µg/kg/h IV to reduce reliance on isoflurane. The common femoral artery was catheterized via direct cut-down technique, which was maintained for monitoring of arterial blood pressure. Systolic blood pressure, heart rate, respiratory rate, end-tidal CO2, oxygen saturation, and core temperature were also closely monitored. Animals were positioned on the operating room table in a prone position, so that the vertex of the skull was facing upwards, and the table tilted to 5° of reverse Trendelenburg (Head up) to ensure adequate venous outflow.

### Surgical technique and instrumentation

Ten scalp fiducials were applied over the cranial convexity of each animal. A pre-operative CT scan was obtained, and the images transferred to the Stealth 7 navigation planning station (Medtronic Navigation, Louisville, CO) for trajectory and registration planning. Trajectories were computed for three separate implants. First, a trajectory was planned for an intracranial electrode/ICP monitor, to be positioned in the deep white matter of the right frontal lobe. The skull entry point for this trajectory was 10 mm anterior to bregma, and 10 mm to the right of midline. The second implant was a 0.15 cc balloon catheter (Edwards Life Sciences, 12A083F), to be used for measuring intracranial compliance. The target location of this implant was the left lateral ventricle, with a skull entry point 10 mm anterior to bregma, and 10 mm to the left of midline. Lastly, a trajectory was planned for a 1.2 cc Fogarty balloon catheter (Edwards Life Sciences, 12TLW806F), to serve as the intracranial lesion. This used a designated skull entry point 7 mm posterior to bregma and 10 mm to the left of midline, with the target lesion in the left parietal lobe 30 mm deep to cortical entry. Specifications of the intracranial instrumentation can be seen in Table 1.

**Table 1:** Specifications for intracranial instrumentation.

Item	Description	Size (F)	Volume (mL)
ME Catheter	Fogarty catheter with thru-lumen for inducing 1) mass effect (ME) through balloon inflation and 2) hemorrhage through blood injection	6	1.2
ICC Catheter	Fogarty catheter placed into left ventricle as direct measurement of intracranial compliance (ICC)	3	0.15
ICP Monitor	Mikro-Tip catheter used for monitoring intracranial pressure (ICP)	3.5	n/a
DBS Electrodes	Intracranial electrodes used for the bioimpedance monitoring system	4.5	n/a

Co-registration of the animals and the pre-operative scan was accomplished using the previously placed scalp fiducials (Fig. 2a) and the AxiEM electromagnetic guidance system (Medtronic Navigation, Louisville, CO). Once registered, three linear scalp incisions corresponding to the planned trajectories were marked, prepped, and draped using standard aseptic technique. The incisions were opened and the underlying skull exposed. Burr holes were fashioned at each of the three sites with a twist drill and rongeurs. The underlying dura was opened and reflected, and a small incision made through the pia-arachnoid membrane.

The three implants were then placed and secured using a custom setup of cranial bolts, angiocatheters, and Touhy–Borst adapters. First, a plastic anchoring bolt was secured into the calvarium in the burr hole. An angiocatheter was then navigated through the bolt to the corresponding target, using a guided stylet. Next, the stylet was removed and replaced with the implant (intracranial electrodes, ICC balloon, or Fogarty balloon). The angiocatheter was withdrawn over the implant, and the setup was secured to the bolt and sealed by closing the Touhy–Borst valves. The scalp incisions around the bolts were packed with petrolatum gauze. Bone wax was administered as needed to ensure any additional outlets were sealed.

Prior to implantation, both the Fogarty and ICC balloon catheters were primed and flushed with dilute contrast solution (20% Visipaque). The thru lumen of the Fogarty catheter used for blood injection was prepped with a 0.6 S/m saline solution (comparable to blood impedance^46^). Saline was used as blood would clot in the time between instrumentation and start of the induced hemorrhage. A postoperative CT scan was obtained to confirm adequate positioning of the implants (Fig. 3g). Specific CT scan parameters can be seen in Supplementary Note 2. The scalp was shaved and prepped with Nuprep electrode gel. Custom templates were used to improve repeatability of electrode positioning. The ICP sensor was calibrated using a three-point procedure before each case.

### Experimental procedure

After instrumentation the pig rested at baseline for 30 min. Following baseline, a linear stage (OEM Syringe Pump, Chemyx Inc., TX) inflated the Fogarty balloon in steps of 100 µL every 5 min to a total volume of 1.2 mL. The stages were controlled through custom serial communication scripts (Matlab, MathWorks, MA). Impedance data from the eight channels and physiologic data from the Biopac system were recorded continuously. The ICC balloon was rapidly inflated to 150 µL volume then deflated every 5 min. CT scans were collected immediately following each 100 µL step in Fogarty inflation, and ICC balloon inflation/deflation occurred 2 min following to ensure the ICC balloon volume was not present at CT. Once fully inflated, the same protocol was used for deflation over a 1-h period. After deflation, 3 mL of autologous blood was removed with a heparin lined syringe. The Fogarty balloon was then pulled back 10 mm to place tip of catheter at the previous balloon site. The blood was then injected in steps of 200 µL every 5 min for 30 min to an equivalent total volume of 1.2 mL. Following blood injection, 100 mL of mannitol was administered. Data was collected during mannitol administration and for 20 min following with CTs recorded every 5 min. Once settled, a lethal dose of Euthasol (Virbac, Cambridge, ON) was administered. BIM and Biopac data collection were then stopped. Blood gasses were collected at the end of baseline, mid-point of inflation, end of inflation, mid-point of deflation, end of deflation, end of blood injection and end of mannitol administration.

### Data exclusions and filtering

All data was pre-processed using the filtering protocol presented in Supplementary Method 1. This filtering removed artifacts and ensured that any impedance change calculated was due to the induced injury and not to factors such as droplets of blood or CSF landing on electrodes (as would frequently occur for the channel directly below the surgical wound). Data not meeting specified quality metrics (as described in Supplementary materials) were excluded. This pre-processing excluded 2/72 baseline traces, 6/72 inflation traces and 7/72 blood injection traces. The 72 traces per event are a product of the number of channels (8) and the number of animals (9). Following filtering, analysis considered two primary attributes of the collected data: change in impedance and change in pressure.

As pigs are live models and subject to variability. Pig 5 blood injection data was excluded due to an internal cerebral herniation resulting in a sudden pressure and impedance drop outside the scope of our induced injury. Additionally, the data for Pig 9 blood injection was only included up to 0.6 mL of volume, as a linear stage serial COM issue resulted in inconsistent injections following 0.6 mL and was not noted until post-procedure data review. However, as all animals were compared by volume, this was adjusted accordingly.

### Data analysis and statistical methods

Statistical significance was set at α = 0.05. Primary data processing and statistical analysis was done in Matlab (R2018b) and JMP Pro (version 15.0.0). Manual image analysis and segmentations of CT scans were performed in Mimics × 64 (version 15.01). No custom code or software was used for processing of image data or JMP Pro statistics.

The individual ten second periods of data collection for each channel were averaged to form a time-series of impedance measurements. The impedances computed from the 80 s prior to the start of inflation was defined as the zero-volume impedance (${Z}_{V0}^{E}$) for each channel, where E is any channel 1–8. For each volume step the average impedance from the 10-s period closest in time to the middle of the 5-min period of static volume was selected as that volume’s Z (${Z}_{Vn}^{E}$), where n is the volume step. This minimized the effect of settling time following inflation and ensured that the ICC balloon was never overlapping the period over which Z is defined. For thoroughness, analysis was done using this 10 s approach, by averaging each full 5 min window or by selecting the 2 min immediately following inflation; results remained robust. Change in Z (ΔZ) was defined as the difference between the final volume impedance ${Z}_{V1.2}^{E}$ and ${Z}_{V0}^{E}$. Inflation data were de-trended by a linear fit extracted from the final 10 min of baseline. This removed any systemic response to initial intracranial instrumentation and helped to isolate the effects of inflation. A potential limitation of this calculation is the dependence on a baseline value (e.g. ${Z}_{V0}^{E}$), however we presume that the clinical acute period of time post-instrumentation represents an equivalently stable baseline. This is similar to the standard clinical monitoring setting in which changes are tracked over time at the bedside, much as ECG, ABP, ICP and other vitals are. Thus, a common scenario can be imagined where the patient has been stabilized, is actively being monitored and then experiences an evolving primary pathology or new secondary pathology.

Pearson’s correlation coefficients between impedance and ICP were computed on both raw and filtered traces, for each element. Volume detection thresholds were defined by the system precision (84 dB SNR) and a safety factor of 10. Single factor t-tests for slope validated consistent blood gas levels and accounted for the small population. To differentiate injury all data was considered to be paired as both injuries occur in the same population. For any paired student’s t-tests all elements within each pig were averaged, creating a single data point per pig. When using all elements, a repeated measures mixed model was selected to allow for multivariate investigation and within-subjects injuries. Subject was set as a Random Effect, Injury and Element set as possible Fixed Effects and Element*Injury considered as a Cross Effect.

Beyond ICV detection and injury differentiation we also explored possible localization. Change in Z over each event (inflation, deflation, hematoma, mannitol, euthanasia) was calculated for each channel within each pig. Within each event for a single pig, the ΔZs were normalized to the maximum element’s ΔZ, creating nΔZs ranging 0–1. Color maps plot the mean nΔZ for each element across all pigs for each event. Of these nΔZ values a mixed model showed ‘element’ to be a significant effect when the event was focal (inflation, deflation and hematoma), and of no effect when global (mannitol, euthanasia). A threshold approach was heuristically determined to analyze an event’s nΔZ values with thresholds of nΔZ < $\frac{1}{3}$ and nΔZ > $\frac{2}{3}$. Should any element satisfy one of these thresholds, the minimum or maximum element was identified as the discrete focal injury element for hemorrhage or model ischemia, respectively. Any event with all nΔZ values between $\frac{1}{3}<{n}\Delta \mathrm{Z}<\frac{2}{3}$ were categorized as global.

While colormaps show promise for localization they are heavily dependent on thresholds. The variance of the element response across all eight electrodes seemed much higher when a focal event occurred than when global (i.e. the response was more heterogeneous). A Levene’s test for equal variance showed significantly unequal variance between focal (inflation and deflation) and global (euthanasia). Levene’s was chosen over alternatives (e.g. Bartlett’s) to be robust to deviations from a normal distribution due to the small sample sizes. To enable comparison of means, var(nΔZ) for the eight channels within a single event were computed for each pig. The single-value-variance has the potential to act as a decision criterion differentiating global from focal. A t-test (Welch’s, chosen to account for unequal variance) showed significant difference between these means. Lastly, an analysis of means (ANOM) looked at the deviation of each event from the group mean by variance, and further differentiated inflation from euthanasia (the two most precise focal and global events).

A statistical limitation of our study is the dual injury model used. Ideally the order of inflation and blood injection would have been randomized in the protocol, however, due to the practical inability to remove the hemorrhage once injected it always occurred second. While non-ideal, the protocol was tightly controlled for consistency across all animals and the primary test chosen (mixed model) does not rely upon the traditional assumption of independent data. Further, while the most ideal model would see separate cohorts for each injury, clinically in a severe trauma case there may be several pathologies occurring (primary trauma, general edema, intracerebral hemorrhage) at once or across the time-scale of secondary monitoring. This may require the BIM to be robust to detecting a mass lesion in-spite of brain swelling, as was the case in this paper. Specifically, the elevation in ICP after balloon deflation (Fig. 3h) to a point above the original baseline value prior to inflation indicates general edema was present following initial injury. While tracking impedance *change* is robust in that it does not require the initial point of injury to match that of a true uninjured baseline, the presence of edema was likely contrasting the hemorrhagic signal and may further explain the variability observed in this injury. While potentially representative of a more complex realistic clinical state, we recognize this as limitation and non-ideal for preliminary quantification. Future work will validate the ability of the BIM to detect ischemic and hemorrhagic injuries in humans, and independent from each other.

## Supplementary Information

Supplementary Informations.

## Data Availability

The data that support the findings of this study are provided in the manuscript, supplementary information and a public repository. All Biopac data are provided at https://figshare.com/s/93db5d71008df3f39d65. All BIM data are provided at https://figshare.com/s/edc2c49513c42471c9d0. CT stacks are available upon request of the corresponding author due to large file size. Any further materials from this study will be provided from the corresponding author upon reasonable request.
