# Supplementary material for: A bioimpedance-based monitor for real-time detection and identification of secondary brain injury
Source: Sci Rep. 2021 Jul 29;11:15454. doi: 10.1038/s41598-021-94600-y (PMC8322167; doi:10.1038/s41598-021-94600-y)
Supplement: Supplementary file 1 — Supplementary Informations. [file 41598_2021_94600_MOESM1_ESM.pdf]

Supplementary Information for

A Bioimpedance-based Monitor for Real-time Detection and Identification of Secondary Brain Injury

Authors: Alicia Everitt*[1], Brandon Root[2], Daniel Calnan[2], Preston Manwaring[3], David Bauer[2] and Ryan Halter[1,2]

Affiliations: [1]Thayer School of Engineering, Dartmouth College, Hanover, NH, 03755. [2]Neurological Surgery, Dartmouth Hitchcock Medical Center, Lebanon, NH, 03766. [3]Rytek Medical, Inc., 16 Cavendish Ct., Lebanon, NH, 03766.

*Corresponding author: Alicia Everitt, email: alicia.c.everitt.th@dartmouth.edu, address: HB 8000, 14 Engineering Dr., Hanover, NH, 03755, phone: 541-399-3643,

Supplementary Table of Contents

Supplementary Table 1: Design specifications for the intracranial bioimpedance monitoring system and testing 4

Supplementary Figure 1: Evaluation of induced CT artifact within gelatin phantoms of varying contrast and materials. **(a)** Alligator clips and DBS with high conductivity phantom (0.6 S/m). **(b)** Alligator clip artifact in a lower conductivity phantom (0.1 S/m). **(c)** CT scans of alternative electrode choices on a gelatin phantom. Radiolucent and tab electrodes yielded the lowest artifact 5

Supplementary Figure 2: Characterization of the developed BIM system showing 100 kHz bandwidth independent of load, mean accuracy of 99.7%, temporal stability of <0.03%, and SNR ~= 85 dB 6

Supplementary Figure 3: Image of 3D printed housing and CT sync circuit 7

Supplementary Figure 4: Key blood gasses show intuitive trends to elevated ICP and no significant confounding anomalies 7

Supplementary Table 2: Summary of all blood gas data acquired across all pigs. Blood gasses were collected using an iStat system at incremental periods during the procedure. One pig had a poorly calibrated iStat with failing cartridges, yielding n=8 for the table below 8

Supplementary Method 1: Expanded Data Filtering 9

Supplementary Figure 5: (Top) Phantom experiment in a saline tank of background conductivity 0.1 S/m tracking each sector as a Fogarty balloon inflates from 0 mL to 5 m in steps. Each sector represents a change in impedance-induced voltage from baseline (V=0). Experiment then repeated with the balloon in a different sector to validate spatial tracking and localization. (Bottom) Saline phantom with continuous inflation of a Fogarty balloon from 0 mL to 3 mL over 15 minutes shows higher resolution volume detect and satisfies design criteria (Supplementary table 1). 14

Supplementary Figure 6: Change in impedance with volume inflation for detrended CT specific elements. As can be observed, there was moderate variability between electrodes, largely due to inherent experimental variables such as the hemostatic ability within subjects, or the varied proximity of the mass effect to the intracranial electrodes. Should it be close to the current sink (intracranial electrodes) its presence would extend beyond a single electrode channel. Vol Detect = 0.39mL+/-.24mL. 15

Supplementary Figure 7: Change in impedance with balloon inflation without adjusting for baseline. While we do not anticipate this scenario clinically while undergoing continuous patient monitoring, should baseline-based detrending not be possible, the BIM still detects an ICV change in 8/9 pigs. Further analysis showed that a single five-minute collection window was sufficient to fit a curve to and detrend electrode drift beyond the change due to injury. Vol Detect = 0.29 mL +/- 0.15 mL. 16

Supplementary Figure 8: ICP alone fails to reject the null of blood injection and inflation being equivalent. Trend differences seen here are likely due to the diffusion of blood by the end of the

injection period, as observed by CT scans. The lower final focal volume of blood would intuitively lead to a lower ICP change, which is seen here. 17

Supplementary Figure 9: DI discriminates between injury types when a single value per pig (all elements averaged)... 18

Supplementary Figure 10: Discrimination based on impedance of high from low 19

Supplementary Figure 11: Axial cross-sections of injury CT scans for every mass effect and hematoma at 1.2 mL (injury completion). Note that this is just a slice of a 3-dimensional inclusion so if diameters seem different this is due to the placement of the slice within the sphere. 19

Supplementary Figure 12: Change in pressure between baseline and two global events: euthanasia and mannitol... 20

Supplementary Figure 13: Unequal variance (p<0.001) between inflation and euthanasia alone 20

Supplementary Figure 14: Comparison of dZ between isolated events inflation and euthanasia show significant unequal variance in 5/9 pigs. 21

Supplementary Note 1: Animal model expanded detail 22

Supplementary Note 2: CT scan parameters.. 23

Supplementary Table 1:

Supplementary Table 1 - Design specifications for the intracranial bioimpedance monitoring system and testing

OBJECTIVES	DESIGN REQUIREMENTS	DESIGN STRATEGY AND ACCEPTANCE CRITERION
(1) Ability to measure small volumes in high noise environment (i.e. ICU)	High signal-to-noise ratio (SNR)	SNR >= 80 dB
		Voltage controlled current source to reduce noise on input power
	Variable frequency	100 kHz bandwidth
		Tetrapolar
	Small volume changes for induced injury	High precision linear stage volume control (.001 uL/min)
	Robust electronics	High input Z amplifiers (CMR above 90 dB at unity gain)
		Low voltage drift (<25 uV/°C)
(2) Ability to detect a focal vs global injury	Multi-sector resolution	8 channels
		Minimum 16 channel capability (two mux)
(3) Ability to differentiate event type	High accuracy	Accuracy >= 99%
		High precision low noise IAs
		Matched filter for voltage sense
	High impedance and low impedance models	Autologous blood injection
		Fogarty balloon inflation
(4) Ability to implement system in a validating surgical environment	Intracranial imaging compatibility (computed tomography)	Fit within bore of a CT scanner
		Ag/AgCl tab electrodes
		Polypropylene cranial bolts
	No additional trauma to 'patient'	ICP coupled to internal electrodes (patented design)
		Biocompatible
	Physiologic monitoring	Biopac vitals
		Blood gasses

Supplementary Figure 1:

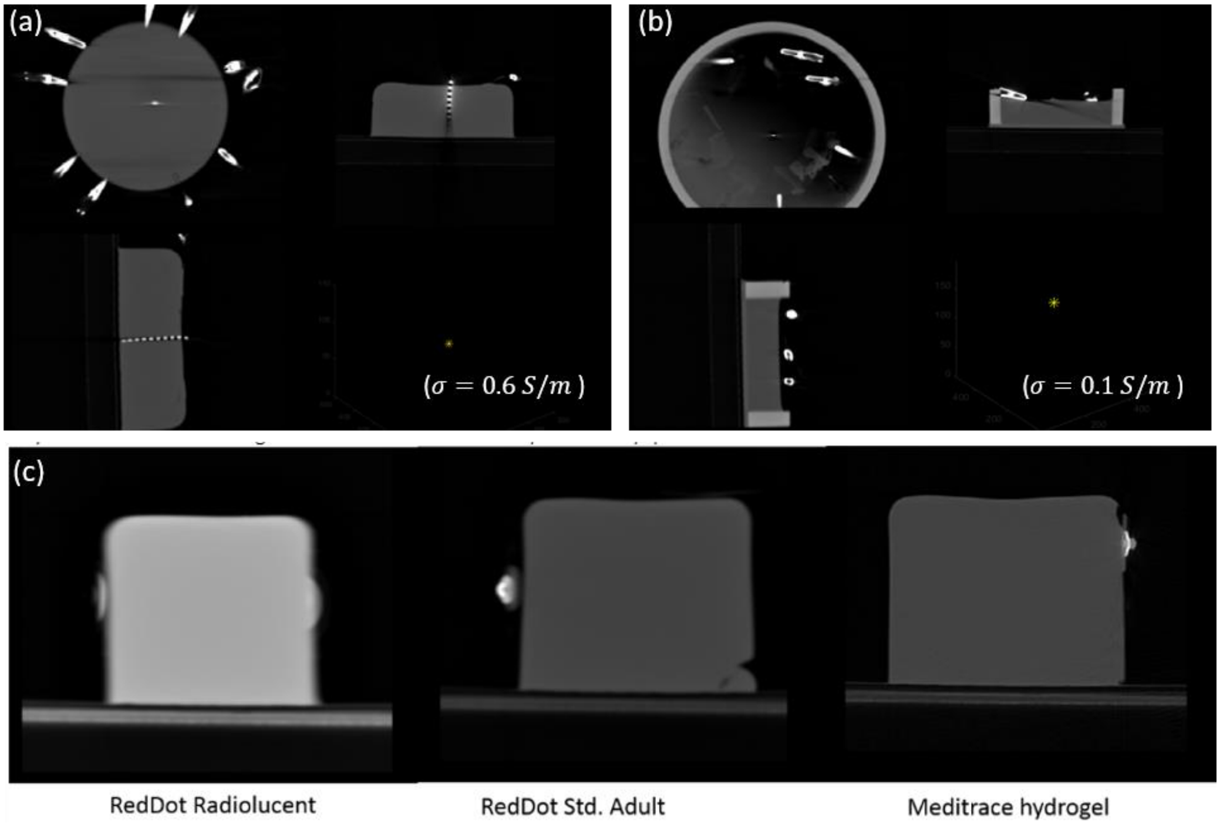

Supplementary Figure 1 –Evaluation of induced CT artifact within gelatin phantoms of varying contrast and materials. **(a)** Alligator clips and DBS with high conductivity phantom (0.6 S/m). **(b)** Alligator clip artifact in a lower conductivity phantom (0.1 S/m). **(c)** CT scans of alternative electrode choices on a gelatin phantom. Radiolucent and tab electrodes yielded the lowest artifact

Supplementary Figure 2

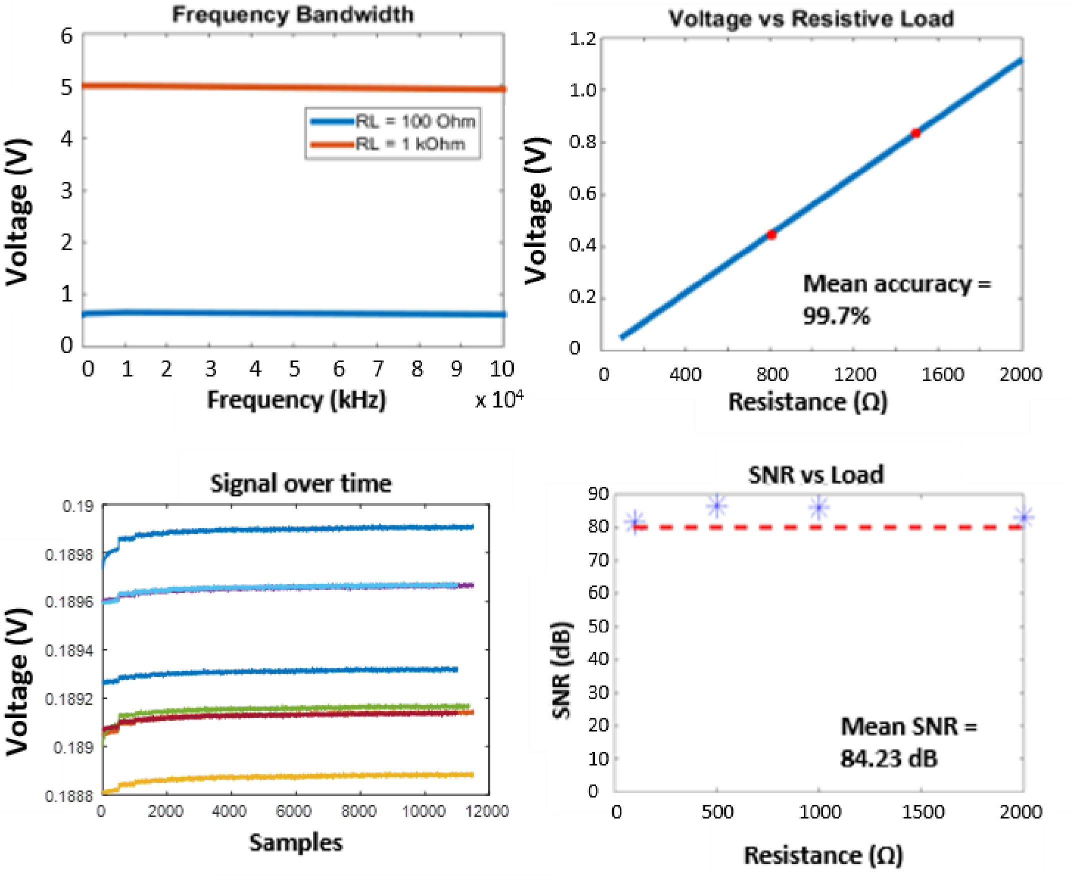

Supplementary Figure 2 - Characterization of the developed BIM system showing 100 kHz bandwidth independent of load, mean accuracy of 99.7%, temporal stability of <0.03%, and SNR ~= 85 dB

Supplementary Figure 3

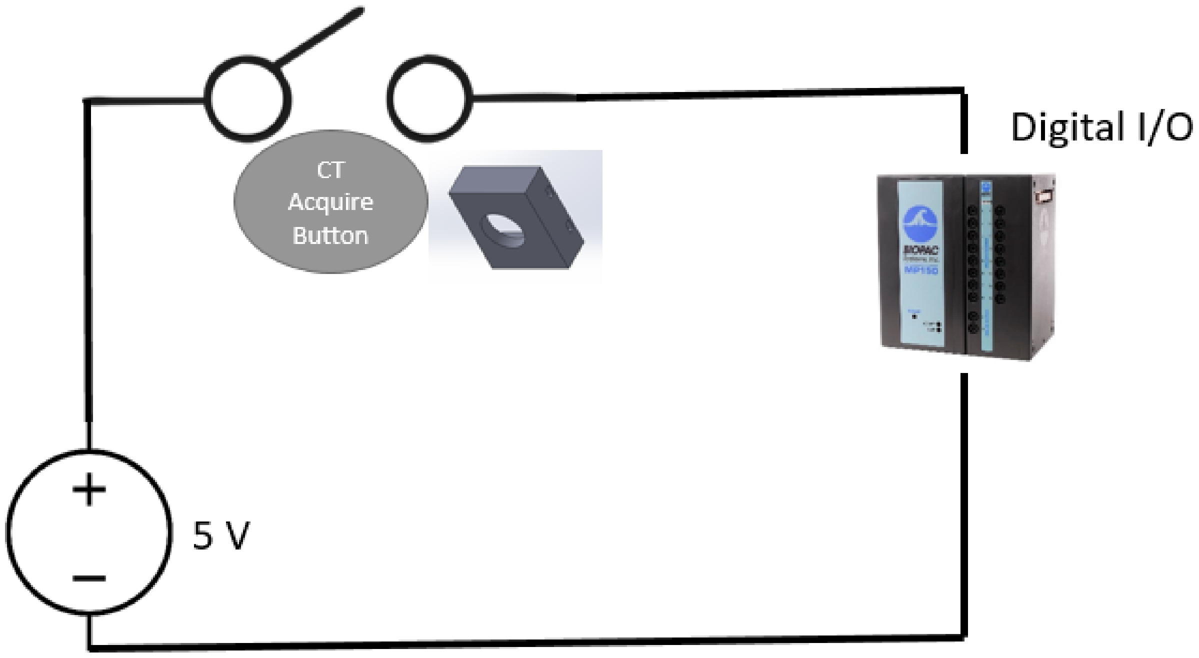

Supplementary Figure 3 - Image of 3D printed housing and CT sync circuit

Supplementary Figure 4

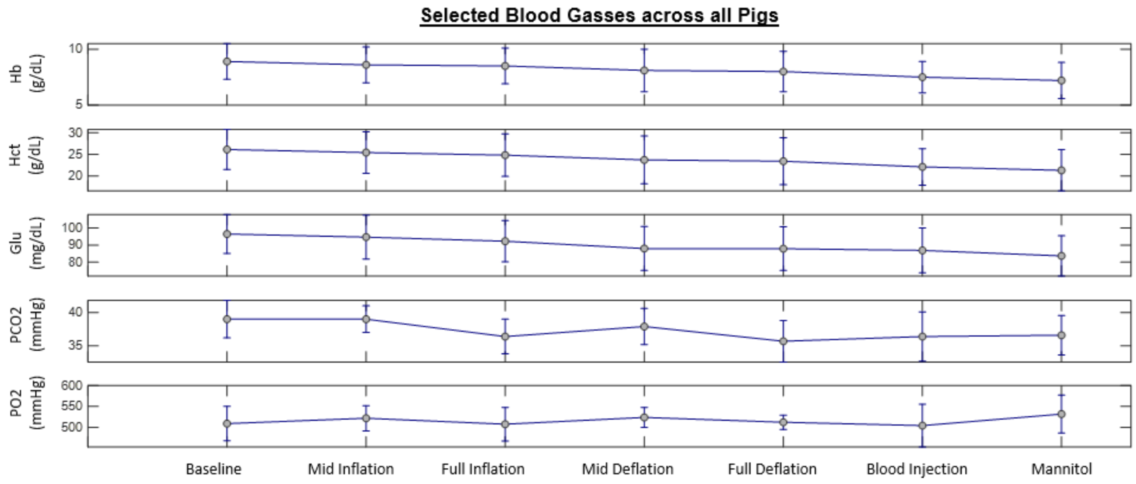

Supplementary Figure 4 - Key blood gasses show intuitive trends to elevated ICP and no significant confounding anomalies

Supplementary Table 2:

Blood gas data is frequently used during surgery or patient monitoring. The summary of all parameters collected can be seen below. Overall the pigs were well ventilated and maintained.

Supplementary Table 2 - Summary of all blood gas data acquired across all pigs. Blood gasses were collected using an iStat system at incremental periods during the procedure. One pig had a poorly calibrated iStat with failing cartridges, yielding n=8 for the table below.

**Parameter**	**Baseline**		**Mid Inflation**		**End of Inflation**		**Mid Deflation**		**End of Deflation**		**Blood Injected**		**Mannitol Administered**	
	Mean	STD	Mean	STD	Mean	STD	Mean	STD	Mean	STD	Mean	STD	Mean	STD
Temp	37	0	37	0	37	0	37	0	37	0	37	0	37	0.00
pH	7.5	0.0	7.5	0.0	7.6	0.0	7.5	0.0	7.6	0.0	7.6	0.1	7.5	0.07
pCO2 mmHg	39.0	2.8	39.0	2.0	36.4	2.6	37.9	2.7	35.7	3.1	36.4	3.7	36.6	2.95
PO2 mmHg	508.8	41.1	521.7	30.2	507.4	40.3	523.6	24.0	512.0	17.1	504.0	51.3	531.6	45.35
BEEcf mmol/L	10.5	2.1	10.9	3.0	10.8	2.7	10.7	3.8	10.8	3.7	10.4	4.3	9.3	4.42
HCO3 mmol/L	32.9	1.7	33.4	2.5	32.9	2.2	33.0	3.3	32.8	3.1	32.6	3.3	31.8	3.29
TCO2 mmol/l	34.0	1.8	34.6	2.3	34.0	2.3	33.9	3.3	33.8	3.2	33.8	3.3	32.9	3.08
sO2%	100	0	100	0	100	0	100	0	100	0	100	0	100	0.00
Na mmol/L	133.3	2.0	132.9	2.0	133.0	2.3	132.0	2.1	132.4	2.2	132.9	2.8	128.7	3.50
K mmol/L	3.8	0.2	3.9	0.2	3.9	0.3	4.0	0.5	3.9	0.5	4.0	0.6	4.0	0.62
iCa mmol/L	1.3	0.0	1.3	0.0	1.3	0.0	1.3	0.0	1.3	0.1	1.2	0.1	1.2	0.07
Glu mg/dl	96.5	11.4	94.7	12.9	92.3	12.1	88.0	12.9	87.9	12.8	86.9	13.1	83.7	11.83
Hct %	26.1	4.6	25.4	4.8	24.8	4.9	23.7	5.5	23.4	5.4	22.1	4.2	21.3	4.75
Hb g/dl	8.9	1.6	8.6	1.6	8.5	1.6	8.1	1.9	8.0	1.8	7.5	1.4	7.2	1.61

Supplementary Method 1

During initial analysis it became apparent that errant electrode traces indicated a need for validation to ensure quality data. A cohesive filtering logic was applied to all the collected raw impedance-induced voltage data unanimously. Primary objectives were to detect faulty electrodes and account for any physiological changes (e.g. a blood drip landing on an electrode). The logic tree for the applied filter can be seen in Supplementary Method 1 Figure 1, and is expanded upon further below.

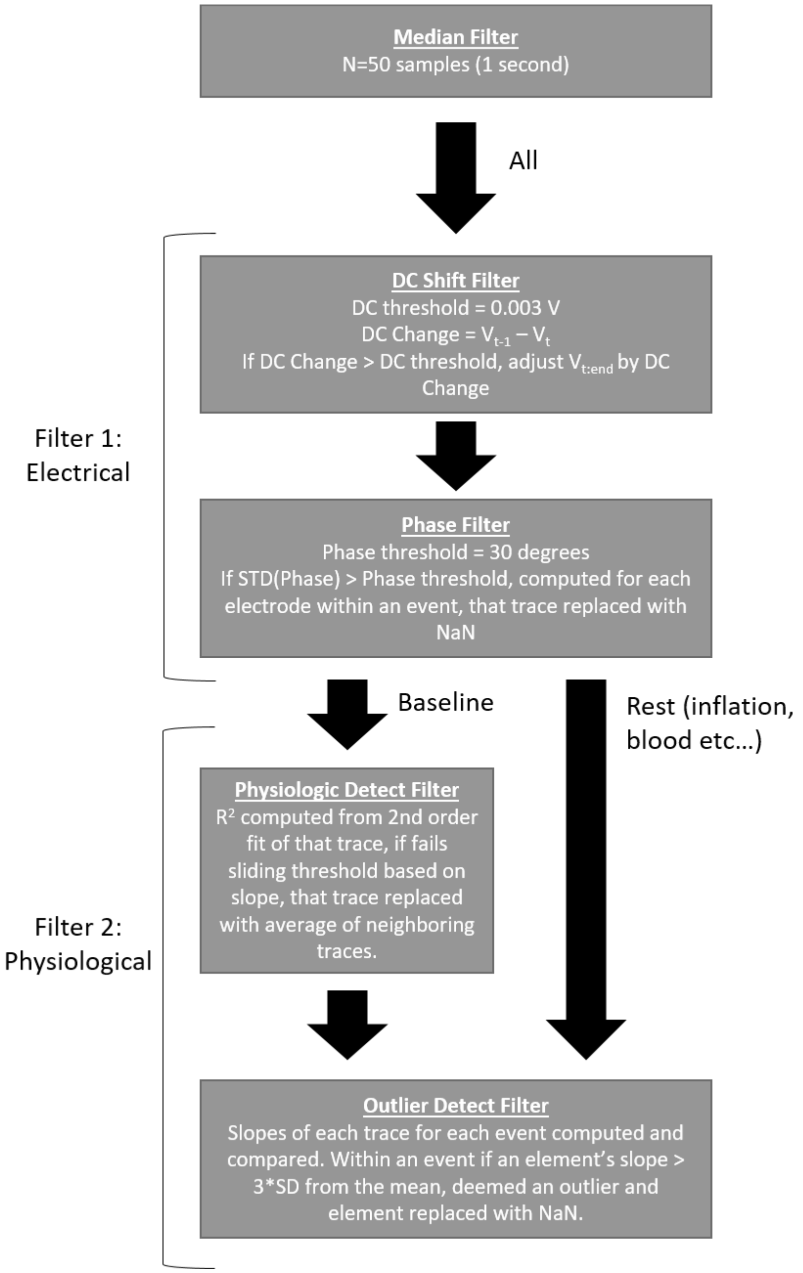

Supplementary Method 1 Figure 1 – Logic tree for filter approach applied to raw impedance data.

First, all data were filtered using a median filter (N=50) to remove CT acquisition noise (Supplementary Method 1 Figure 2, left). The first level filter logic was an electrical filter which included a DC shift filter and phase filter. While voltage traces represented the dependent variable, phase factored in the stimulating current. An example of the DC filter followed by a phase identified trace example can be seen in Supplementary Method 1 Figures 2 and 3.

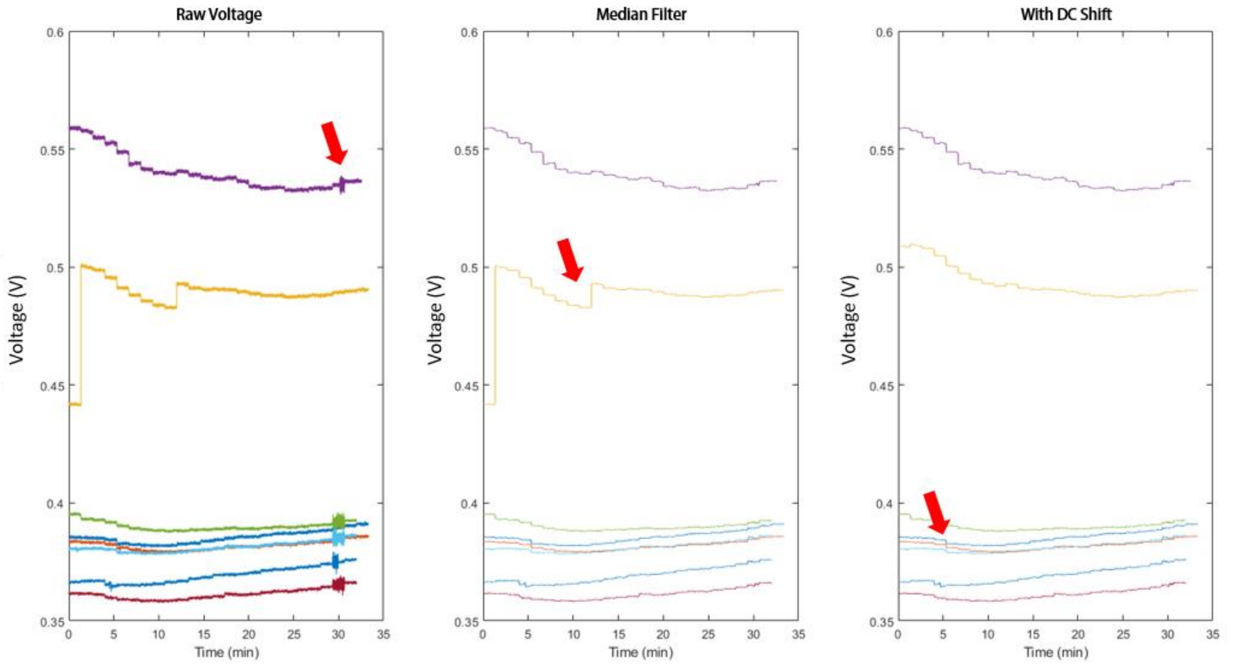

Supplementary Method 1 Figure 2 – All element baseline data for Pig 7 in raw form (left), with median filter applied (middle) and with DC filter applied (right). Example of Median filter removing the CT acquisition noise (farthest left red arrow), a large DC shift (middle red arrow) and a small DC shift not removed due to coarseness of threshold for DC detection (farthest right red arrow).It is important to remember that each of these eight traces were collected sequentially, so while displayed against time on the x-axis, each collection on each channel had the sequential channels collect before it's next sampling.

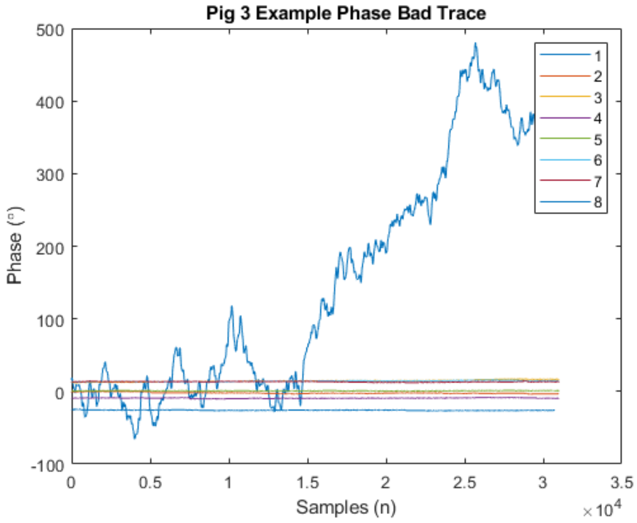

Supplementary Method 1 Figure 3 - Example bad trace in Pig 3 by phase filter

Due to the high noise environment of the CT bore and linked grounds from all systems with the AFE, the impedance traces would experience occasional DC shifts (Supplementary Figure 2, middle). To fairly represent the changes in impedance due to the injury, and not due to a digital signal jump, a filter was applied to subtract any sudden change larger than a specified voltage threshold. The threshold was determined heuristically and the filter applied across all data unanimously.

An example of a trace which the DC filter identified and corrected can be seen in Supplementary Method 1 Figure 2. In-spite of these shifts the impedance trends would persist, however the shifts falsely inflated our changing Z if not removed. Large shifts were successfully corrected using the DC filter. Small steps (e.g. Supplementary Method 1 Figure 2, right), if under the threshold, would remain. A tighter threshold would capture these, however, small noise changes may not always be correctable in clinic. Additionally, the DC filter code looked at changes between two time-steps (Z_{t2}-Z_{t1}, fs = 50 Hz). This will fail to catch any more gradual changes, however if a confounding gradual change was riding on an electrode this would not qualify as a DC shift, and if an errant electrode, should be caught in the following filter step. For these reasons we chose the more conservative coarser filter threshold, used single time-steps, and found our data trends to be robust in-spite of these small anomalies.

While filter one corrected for electrically-induced variations, filter two (i.e. Physiological Detect Filter) aimed to identify physiologic changes. In the second filter step all baseline traces were fit to a second order polynomial and subject to an R^2 based goodness-of-fit filter. The mean R^2 was higher for a second order polynomial than a first order one, suggesting this to be a more representative fit. Supplementary Method 1 Figure 4 shows an example of such a trace. Upon review of photos it was apparent that the element identified had CSF under the electrode, however, after a settling time, fluid compromised electrodes can still provide valuable information on changing impedance. Additionally, during baseline all traces tend to settle similarly unless acted upon otherwise (e.g. blood drip). Thus, if a baseline trace was identified as failing the polynomial fit, that trace would be replaced with an average of the neighboring elements. In further processing, should this electrode fail any injury event filters it would be discarded.

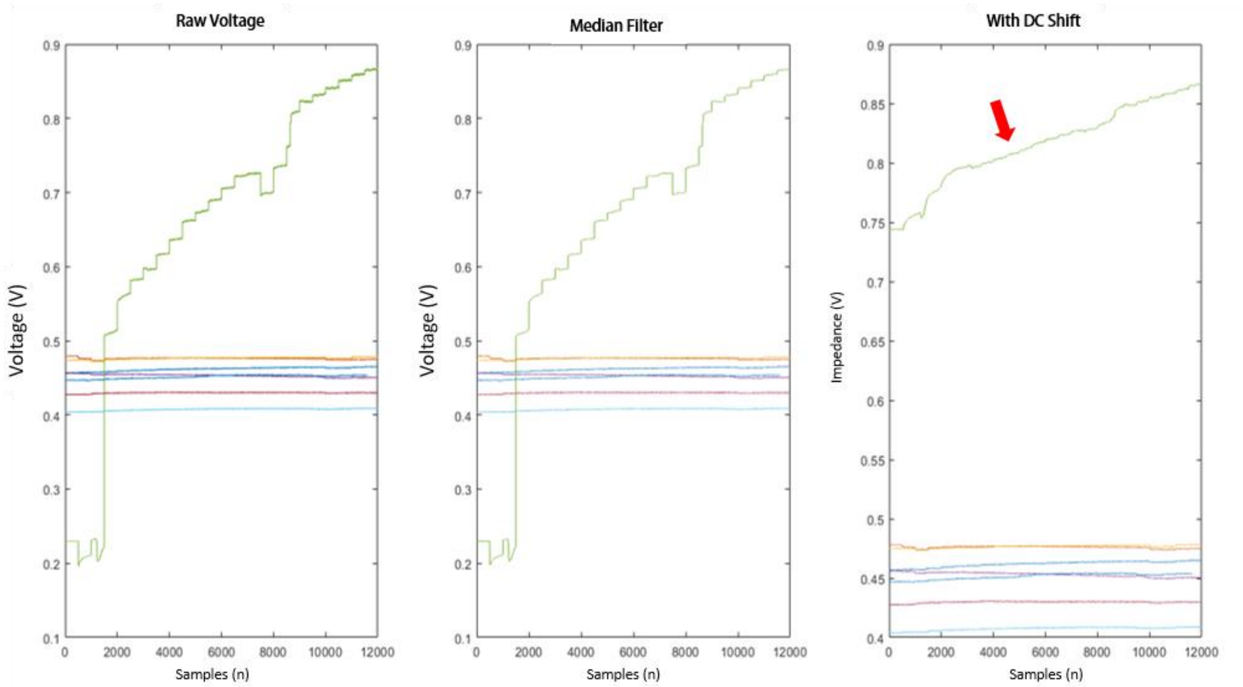

Supplementary Method 1 Figure 4 - Baseline data with a blood affected electrode (red arrow). This element was identified by the R^2 filter applied to baseline data.

Lastly, the Physiologic Filter was applied to all data and used to detect outliers. A second order polynomial was fit to each trace within each event (e.g. baseline, inflation, blood). An outlier was defined as any element whose slope was more than three standard deviations away from the mean of all the elements within that event (Supplementary Method 1 Figure 5). Once identified as an outlier, these elements were discarded.

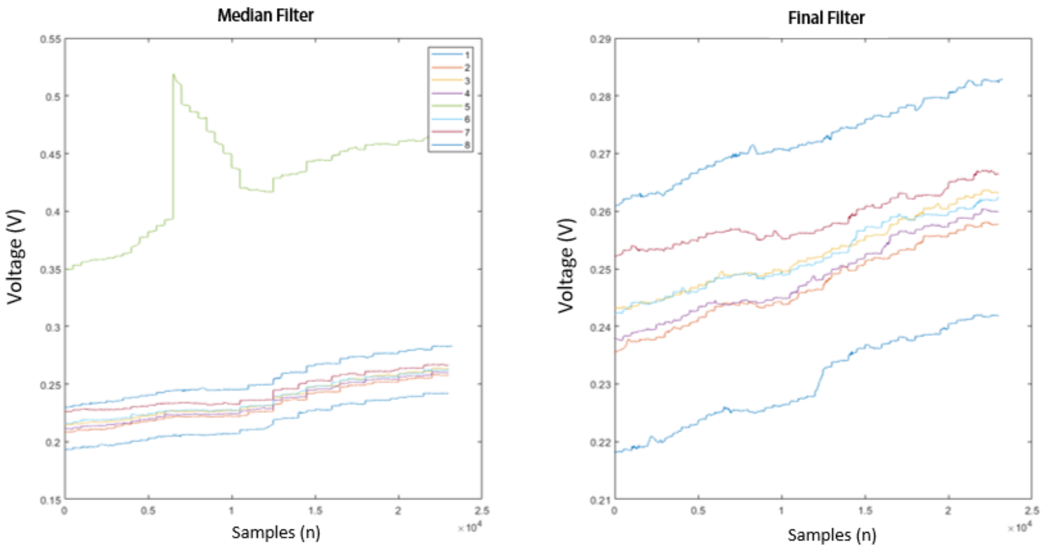

Supplementary Method 1 Figure 5 - Pig 8 inflation data. All traces after median filter (left) and all traces after full filter logic (right). Note that element 5 has been identified as an outlier and discarded.

The summary numbers of elements affected by the filtering protocol can be seen in Supplementary Method 1 Table 1 below. The element specific details split by pig for each of these filtered elements can be seen in Supplementary Method 1 Table 2 below. Elements 3-5 are the most frequently affected due to their location proximal to the larger surgical incision, which was prone to leaking until hemostasis was accomplished.

Supplementary Method 1 Table 1 - Number summary of elements affected by filtering (i.e. replaced with NaN)

	Number of Traces Flagged	Total Number of Traces	Percent Removed (%)
**Baseline**	2	72	2.78
**Inflation**	6	72	8.33
**Blood**	7	72	9.72

Supplementary Method 1 Table 2 - Affected elements (noted as NaN) within each pig and each event for the applied filter algorithm.

**Baseline**	Pig 1	El 1	El 2	El 3	El 4	El 5	El 6	El 7	El 8
	Pig 2	El 1	El 2	El 3	**NaN**	El 5	El 6	El 7	El 8
	Pig 3	El 1	El 2	El 3	El 4	El 5	El 6	El 7	El 8
	Pig 4	El 1	El 2	El 3	El 4	El 5	El 6	El 7	El 8
	Pig 5	El 1	El 2	El 3	El 4	El 5	El 6	El 7	El 8
	Pig 6	El 1	El 2	El 3	El 4	El 5	El 6	El 7	El 8
	Pig 7	El 1	El 2	**NaN**	El 4	El 5	El 6	El 7	El 8
	Pig 8	El 1	El 2	El 3	El 4	El 5	El 6	El 7	El 8
	Pig 9	El 1	El 2	El 3	El 4	El 5	El 6	El 7	El 8
**Inflation**	Pig 1	El 1	El 2	El 3	El 4	El 5	El 6	El 7	El 8
	Pig 2	El 1	El 2	El 3	**NaN**	El 5	El 6	El 7	El 8
	Pig 3	**NaN**	El 2	El 3	El 4	**NaN**	El 6	El 7	El 8
	Pig 4	El 1	El 2	El 3	El 4	El 5	El 6	El 7	El 8
	Pig 5	El 1	El 2	El 3	El 4	El 5	El 6	El 7	El 8
	Pig 6	El 1	El 2	El 3	El 4	**NaN**	El 6	El 7	El 8
	Pig 7	El 1	El 2	El 3	El 4	El 5	El 6	El 7	El 8
	Pig 8	El 1	El 2	El 3	El 4	**NaN**	El 6	El 7	El 8
	Pig 9	El 1	El 2	**NaN**	El 4	El 5	El 6	El 7	El 8
**Blood**	Pig 1	El 1	El 2	El 3	El 4	El 5	El 6	El 7	El 8
	Pig 2	El 1	El 2	El 3	El 4	El 5	El 6	El 7	El 8
	Pig 3	**NaN**	**NaN**	El 3	El 4	El 5	El 6	El 7	El 8
	Pig 4	El 1	El 2	El 3	El 4	El 5	El 6	El 7	El 8
	Pig 5	El 1	El 2	El 3	El 4	El 5	El 6	El 7	El 8
	Pig 6	El 1	El 2	El 3	El 4	**NaN**	El 6	El 7	El 8
	Pig 7	El 1	El 2	El 3	El 4	**NaN**	El 6	El 7	El 8
	Pig 8	El 1	El 2	**NaN**	El 4	El 5	El 6	El 7	El 8
	Pig 9	El 1	**NaN**	**NaN**	El 4	El 5	El 6	El 7	El 8

Supplementary Figure 5

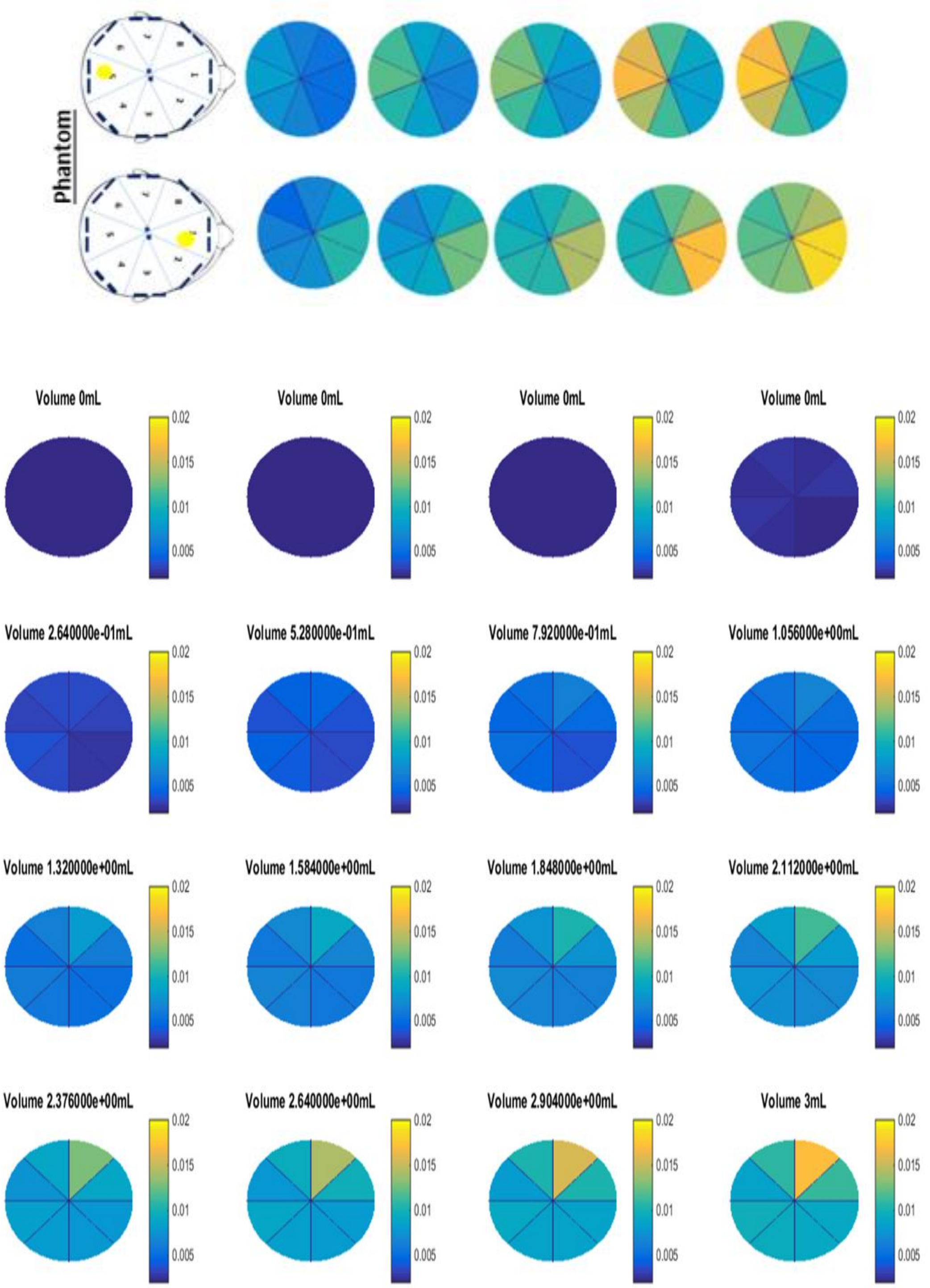

Supplementary Figure 5 – (Top) Phantom experiment in a saline tank of background conductivity 0.1 S/m tracking each sector as a Fogarty balloon inflates from 0 mL to 5 m in steps. Each sector represents a change in impedance-induced voltage from baseline (V=0). Experiment then repeated with the balloon in a different sector to validate spatial tracking and localization. (Bottom) Saline phantom with continuous inflation of a Fogarty balloon from 0 mL to 3 mL over 15 minutes shows higher resolution volume detect and satisfies design criteria (Supplementary table 1).

Supplementary Figure 6:

Impedance detects change in intracranial volume in 9/9 pigs using a priori injury scan.

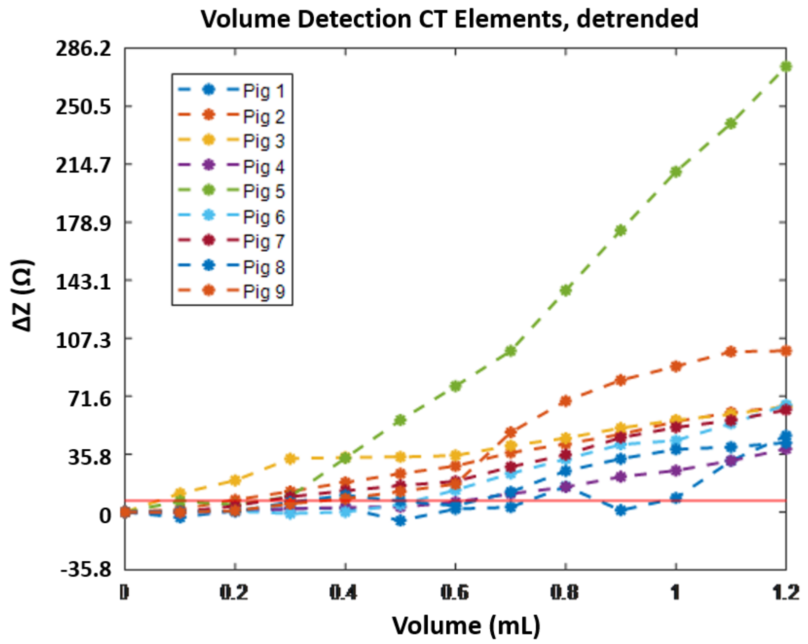

Supplementary Figure 6 - Change in impedance with volume inflation for detrended CT specific elements. As can be observed, there was moderate variability between electrodes, largely due to inherent experimental variables such as the hemostatic ability within subjects, or the varied proximity of the mass effect to the intracranial electrodes. Should it be close to the current sink (intracranial electrodes) its presence would extend beyond a single electrode channel. Vol Detect = 0.39mL+/-.24mL.

Supplementary Figure 7:

Impedance detects change in intracranial volume in 8/9 pigs without any baseline adjustment.

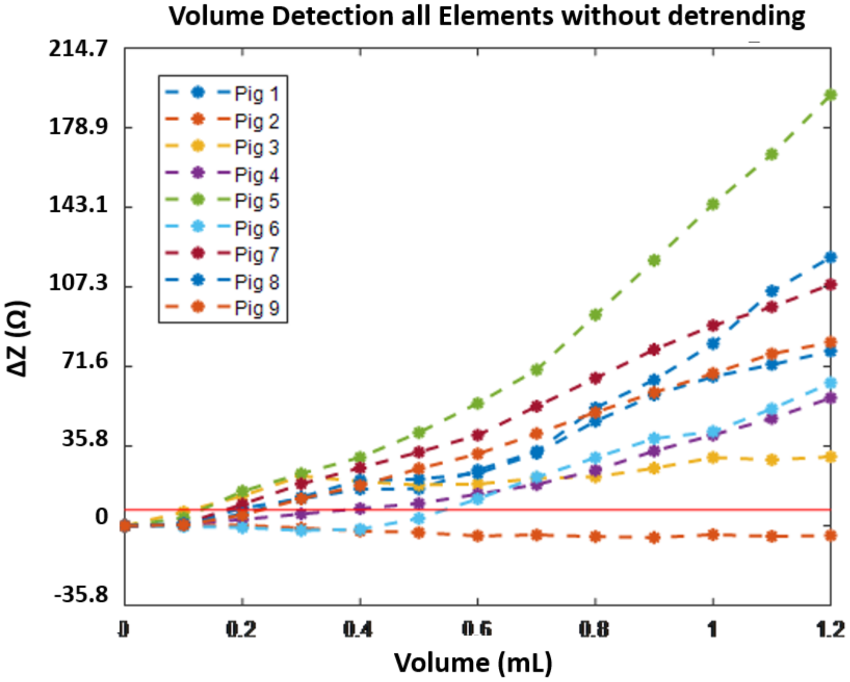

Supplementary Figure 7 - Change in impedance with balloon inflation without adjusting for baseline. While we do not anticipate this scenario clinically while undergoing continuous patient monitoring, should baseline-based detrending not be possible, the BIM still detects an ICV change in 8/9 pigs. Further analysis showed that a single five-minute collection window was sufficient to fit a curve to and detrend electrode drift beyond the change due to injury. Vol Detect = 0.29 mL +/- 0.15 mL.

Supplementary Figure 8:
ICP alone does not differentiate between injury types

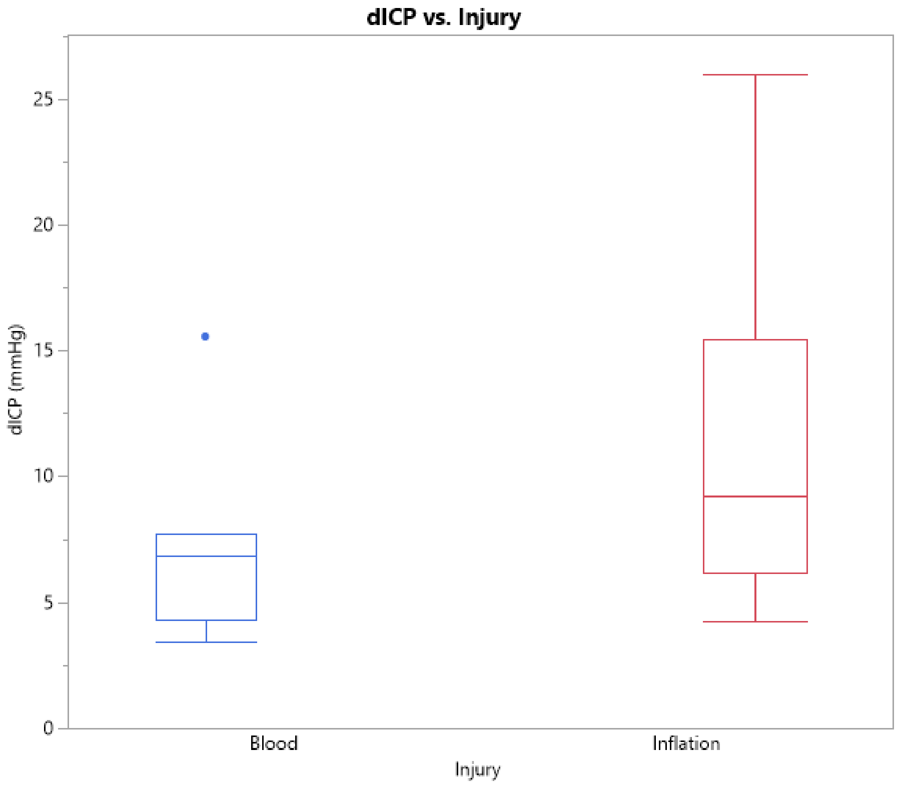

Supplementary Figure 8 – ICP alone fails to reject the null of blood injection and inflation being equivalent. Trend differences seen here are likely due to the diffusion of blood by the end of the injection period, as observed by CT scans. The lower final focal volume of blood would intuitively lead to a lower ICP change, which is seen here.

Supplementary Figure 9:

If all elements are averaged together per pig, a paired student’s t-test significantly differentiates between injury types.

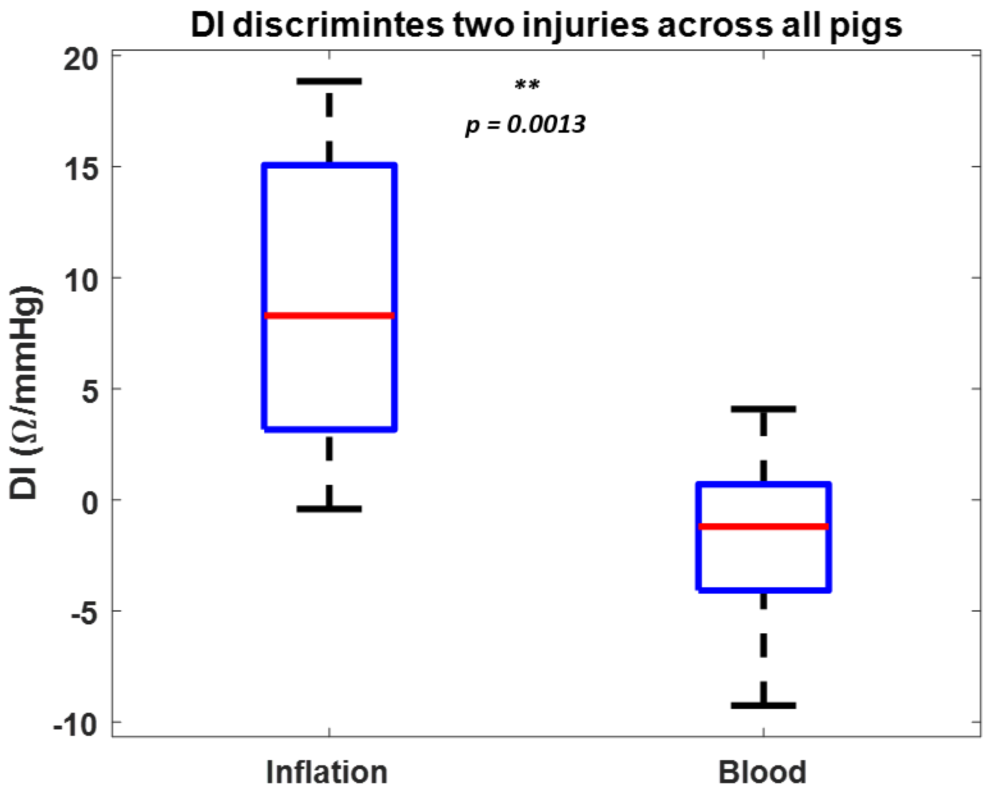

Supplementary Figure 9 - DI discriminates between injury types when a single value per pig (all elements averaged).

Supplementary Figure 10:

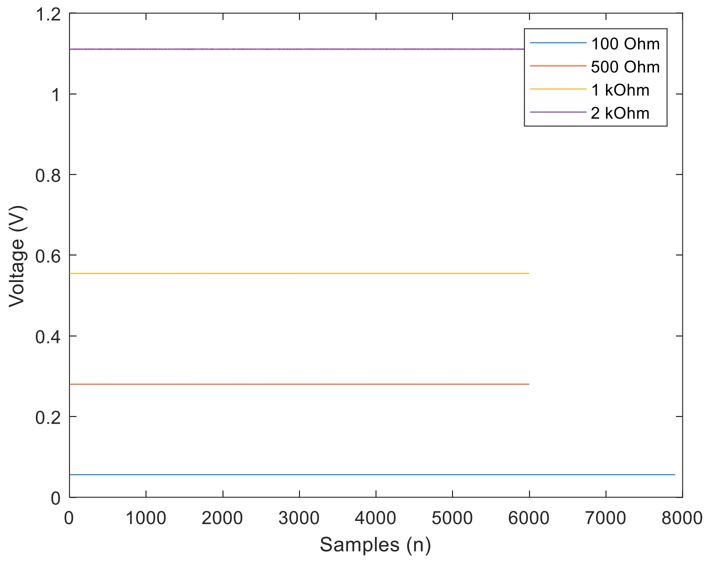

Supplementary Figure 10 - Discrimination based on impedance of high from low

Supplementary Figure 11:

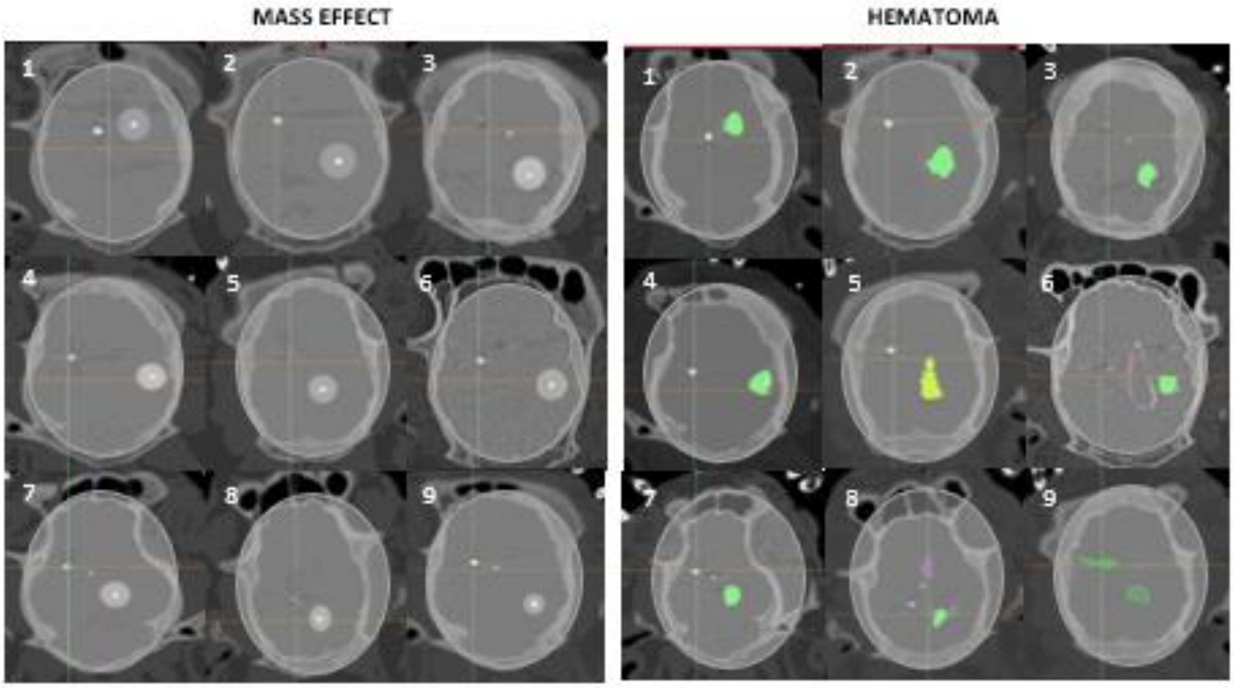

Supplementary Figure 11 - Axial cross-sections of injury CT scans for every mass effect and hematoma at 1.2 mL (injury completion). Note that this is just a slice of a 3-dimensional inclusion so if diameters seem different this is due to the placement of the slice within the sphere.

Supplementary Figure 12

ICP detects both euthanasia and mannitol as “ICV” events when compared to baseline ($p < 0.001$, both), however does not differentiate them from each other.

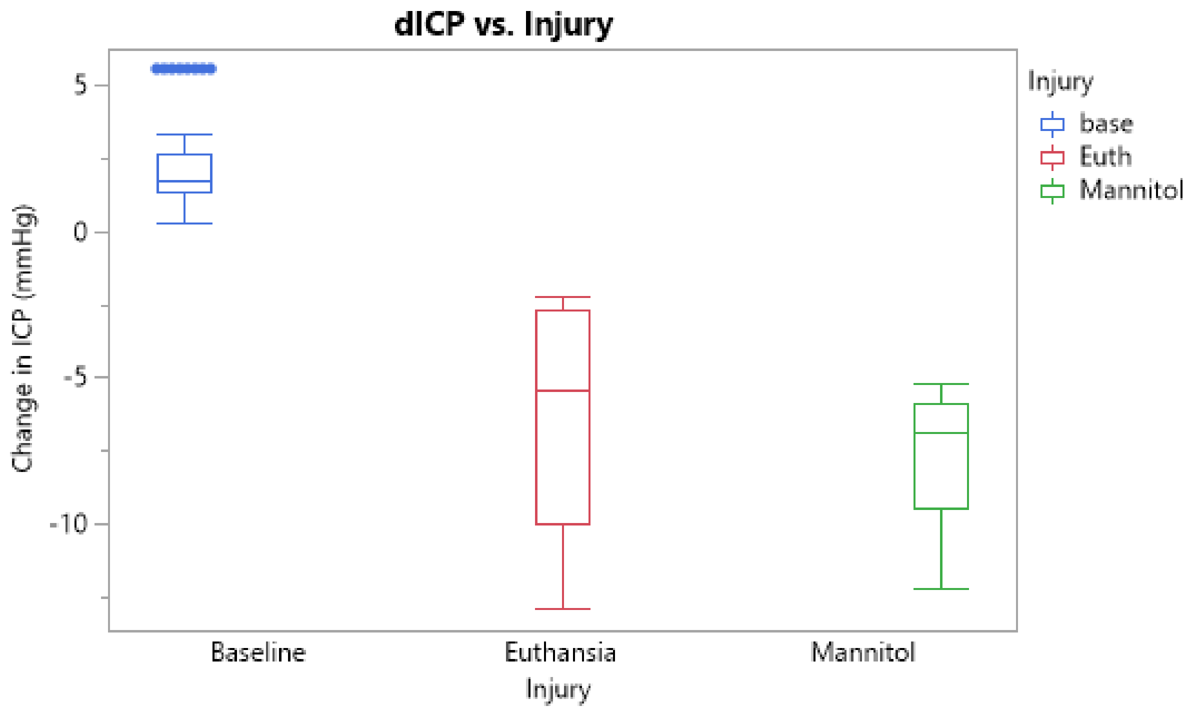

Supplementary Figure 12 - Change in pressure between baseline and two global events: euthanasia and mannitol

Supplementary Figure 13:

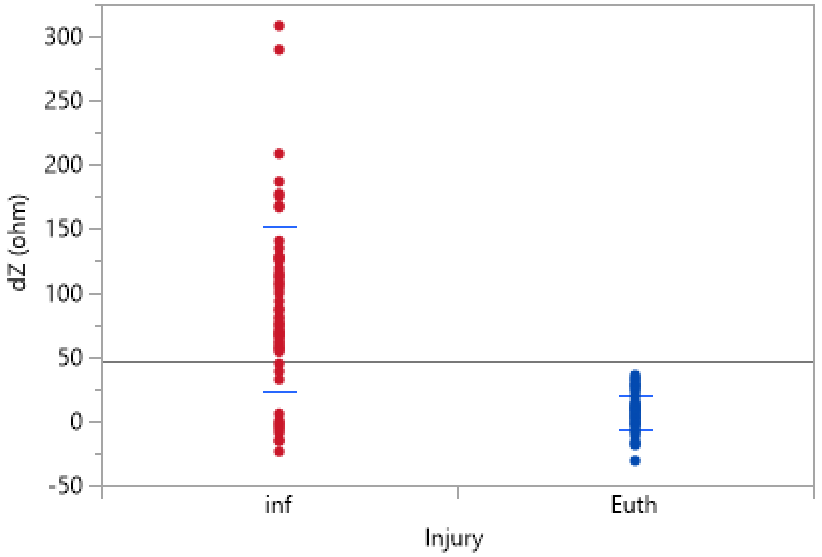

Supplementary Figure 13 - Unequal variance ($p<0.001$) between inflation and euthanasia alone

Supplementary Figure 14:

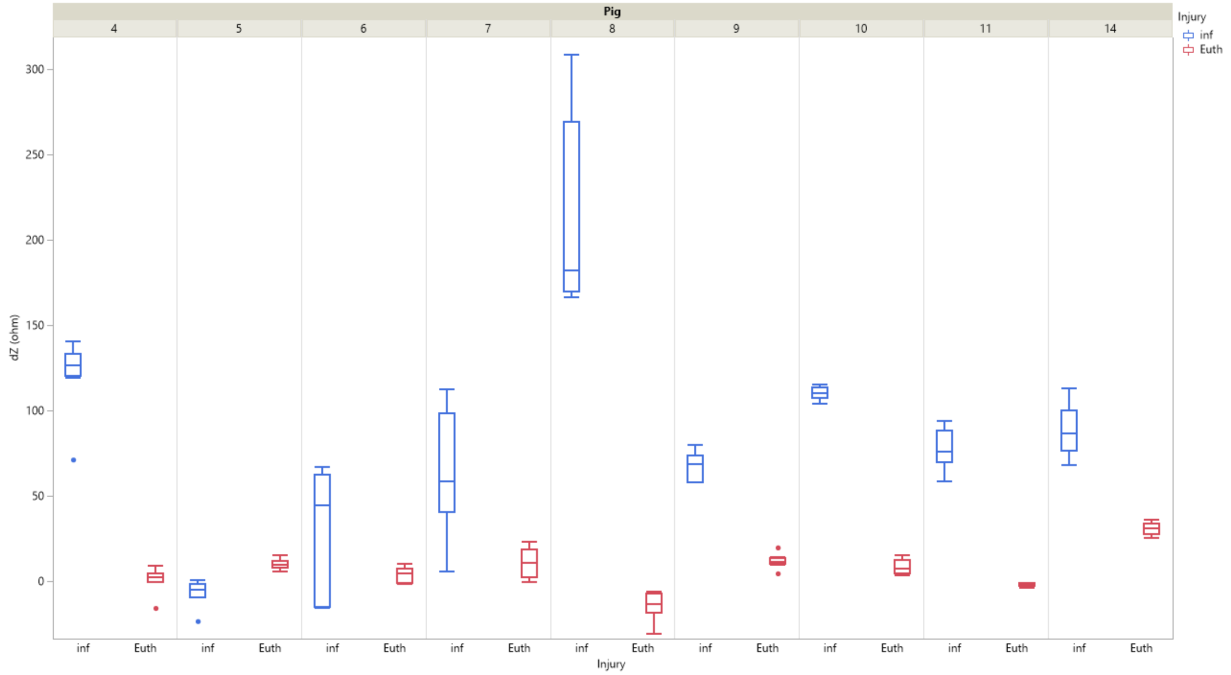

Supplementary Figure 14 - Comparison of dZ between isolated events inflation and euthanasia show significant unequal variance in 5/9 pigs.

Supplementary Note 1:

When considering which animal to select, many have used smaller animal species such as rodents, largely due to ease of use and low cost, however differences in their neural morphology and pathology have demonstrated necessity of larger animals[1–3]. Specifically, both humans and larger mammals, such as pigs, have gyrencephalic brains while rats and mice have lissencephalic brains and different white to grey matter ratios[4,5]. Xiong et al. states in Nature Reviews Neuroscience that "it is extremely important to further develop and increasingly use higher species with brains that are more anatomically and functionally closer to man[2]" in regards to brain injury research. While this can indicate any large mammal—such as swine, sheep, dogs or monkeys—pigs are well established as a model for brain injury and allow morphological benefits while still respecting the phylogenetic scale[6–8].

1. Morganti-Kossmann MC, Yan E, Bye N. Animal models of traumatic brain injury: Is there an optimal model to reproduce human brain injury in the laboratory? Injury. 2010;41:S10-S13. doi:10.1016/j.injury.2010.03.032

2. Xiong Y, Mahmood A, Chopp M. Animal models of traumatic brain injury. Nat Rev Neurosci. 2013;14(2):128-142. doi:10.1038/nrn3407

3. Duhaime A-C. Large Animal Models of Traumatic Injury to the Immature Brain. Dev Neurosci. 2006;28(4-5):380-387. doi:10.1159/000094164

4. Cullen DK, Harris JP, Browne KD, et al. A Porcine Model of Traumatic Brain Injury via Head Rotational Acceleration. In: Methods in Molecular Biology (Clifton, N.J.). Vol 1462. ; 2016:289-324. doi:10.1007/978-1-4939-3816-2_17

5. Pareja JCM, Keeley K, Duhaime A-C, Dodge CP. Modeling Pediatric Brain Trauma: Piglet Model of Controlled Cortical Impact. In: Humana Press, New York, NY; 2016:345-356. doi:10.1007/978-1-4939-3816-2_19

6. Fritz HG, Walter B, Holzmayr M, Brodhun M, Patt S, Bauer R. A Pig Model with Secondary Increase of Intracranial Pressure after Severe Traumatic Brain Injury and Temporary Blood Loss. J Neurotrauma. 2005;22(7):807-821. doi:10.1089/neu.2005.22.807

7. Purins K, Sedigh A, Molnar C, et al. Standardized experimental brain death model for studies of intracranial dynamics, organ preservation, and organ transplantation in the pig*. 2011. doi:10.1097/CCM.0b013e318206b824

8. Andrade AF de, Soares MS, Patriota GC, et al. Experimental model of intracranial hypertension with continuous multiparametric monitoring in swine. Arq Neuropsiquiatr. 2013;71(10):802-806. doi:10.1590/0004-282X20130126

Supplementary Note 2:

<u>Extrapolation of the CT scan parameters and protocol</u>

1. Topogram to survey anatomy of interest – mA 40 / kV 120, slice 0.6 mm
2. Pre Monitoring at the ascending aorta – mAs 40 / kV 120, slice 10 mm
3. Contrast Administration 5 ml/s flow rate – Test Bolus 20 ml of saline
4. 30 ml iodinated contrast "Visipaque" 320 mgI/ml followed by 40 ml saline flush
5. Monitoring at the ascending aorta – mAs 40 / kV 120, slice 10 mm, auto triggering arterial scan at 101 E(HU)
6. Head Arterial – mAs 280 / kV 120, slice 1 mm, pitch 0.95
7. Head Venous – scan delay 25 second, mAs 280 / kV 120, slice 1 mm, pitch 0.95
8. Head for Stealth Nav – mAs 380 / kV 120, slice 0.6 mm, pitch 1
